# HER2/*ERBB2* and Genomic Alterations in Patients with Gallbladder Cancer from Argentina

**DOI:** 10.3390/cancers18213461

**Published:** 2026-10-27

**Authors:** Gerardo F. Arroyo, Gabriela I. Acosta Haab, Aylén Avila, Oluseyi B. Abidoye, Elizabeth A. Raupach, Esteban Braggio, Mitesh J. Borad

**Affiliations:** 1 ILOGI (Latin American Intergroup of Gatrointestinal Oncology), San Salvador de Jujuy 4600, Jujuy, Argentina; 2 San Isidro Patología, San Isidro 1642, Provincia de Buenos Aires, Argentina; gabrielaacostahaab@gmail.com; 3 Centro de Investigación del Cáncer Rosario y Cox Analíticas, Rosario 2000, Santa Fe, Argentina; aylen.avila@gmail.com; 4 Department of Hematology-Oncology, Mayo Clinic Cancer Center, Phoenix, AZ 85054, USA; abidoye.oluseyi@mayo.edu (O.B.A.); raupach.elizabeth@mayo.edu (E.A.R.); braggio.esteban@mayo.edu (E.B.); borad.mitesh@mayo.edu (M.J.B.)

**Keywords:** HER2/*ERBB2*, genomic abnormalities, gene sequencing variants, gallbladder cancer, Argentina

## Abstract

Background/Objectives: Gallbladder cancer (GBC) is highly prevalent in Western Argentina, where the frequency and clinical significance of HER2/*ERBB2* abnormalities remain unknown. We investigated the prevalence, prognostic impact, and genomic co-occurrence of HER2/*ERBB2* alterations in Argentine patients with GBC. Methods: We retrospectively analyzed patients with GBC from the Latin American Intergroup of Gastrointestinal Oncology (ILOGI) database. HER2 protein expression was assessed by immunohistochemistry, and sequence variants in *ERBB2* and 213 other cancer-related genes by next-generation sequencing. Overall survival (OS) was analyzed using Kaplan–Meier curves, the log-rank test, and univariate Cox proportional hazards regression. Genomic co-occurrences were evaluated using Fisher’s exact test. Results: Thirty patients were included in the study. Most were women with gallstones and advanced-stage disease. HER2 overexpression was detected in five patients (17%), with *ERBB2* variants in three other tumors, yielding an overall HER2/*ERBB2* alteration rate of 27% (8/30). Among patients with stage III–IV disease, HER2/*ERBB2* abnormalities were not associated with a statistically significant difference in OS (HR, 0.40; 95% CI, 0.11–1.45; *p* = 0.162). Similarly, HER2 overexpression alone had no significant prognostic impact. Exploratory analysis identified potential co-occurrence of HER2/*ERBB2* abnormalities with sequence variants in 16 genes involved in chemotherapy sensitivity and the PI3K/AKT/mTOR and VEGF signaling pathways, although these associations were not statistically significant after correction for multiple comparisons. Conclusions: HER2/*ERBB2* abnormalities are frequent among Argentine patients with GBC and do not appear to be associated with poorer OS. Our findings provide a biologic rationale for further investigation of the potential synergistic effects of combining anti-HER2 therapies with PI3K/AKT/mTOR or VEGF pathway inhibitors.

## Simple Summary

Gallbladder cancer (GBC) is a significant health burden in northwestern Argentina, where it is the third leading cause of cancer-related death among women. Given the prognostic relevance of HER2 abnormalities and their potential to predict response to effective, available anti-HER2 therapies, our study aimed to determine the prevalence and prognostic significance of HER2/*ERBB2* abnormalities and to characterize their co-occurring genomic alterations in patients with GBC from northwestern Argentina, thereby contributing to a better understanding of this important regional health problem. HER2/*ERBB2* abnormalities were identified in 27% of patients and were not associated with poorer overall survival. These abnormalities showed potential co-occurrence with genomic alterations involving the PI3K/AKT/mTOR and VEGF signaling pathways. Studies in larger cohorts are needed to validate the prevalence and prognostic role of HER2/*ERBB2* abnormalities and to investigate potential synergy between HER2-targeted agents and inhibitors of the PI3K/AKT/mTOR or VEGF signaling pathways.

## 1. Introduction

Gallbladder cancer (GBC) is an uncommon malignancy globally, but carries striking geographic heterogeneity in incidence, with the highest burdens observed in parts of South America and Asia [1,2,3]. Despite incremental advances in systemic therapy for biliary tract cancers, outcomes for GBC remain poor, reflecting late presentation, limited therapeutic targets, and a scarcity of disease-specific evidence [4]. Molecular profiling has begun to identify subsets of biliary tract cancers with actionable alterations, including *ERBB2* (HER2) amplification, overexpression, and activating mutations [5]. Although HER2-directed therapies have transformed the management of HER2-positive breast and gastric cancers, the prevalence, spectrum, and clinical significance of HER2 abnormalities in GBC vary considerably across studies and regions, and their prognostic impact remains uncertain.

Across published series encompassing different biliary tract cancer sites, HER2 alterations have been reported most frequently in extrahepatic cholangiocarcinoma and GBC [6,7]. However, estimates of HER2 positivity in GBC vary broadly [8], likely due to small sample sizes, differing histologic composition, and methodological variability (e.g., immunohistochemistry [IHC] scoring criteria, in situ hybridization, and next-generation sequencing [NGS] panels). Emerging reports of clinical activity with HER2-directed regimens [4,5,6,8,9] such as trastuzumab-based combinations, pertuzumab, and antibody-drug conjugates, in HER2/*ERBB2*-altered biliary tract cancers underscore the need for robust, site-specific characterization of HER2 as both a biomarker and a therapeutic target in GBC.

Argentina includes regions with a high mortality of GBC within the Andean belt [10]. In the northwestern provinces of Salta and Jujuy, where GBC is a major public health concern, it has been reported to be the third leading cause of cancer-related mortality among women [11]. In such settings, defining the molecular landscape is particularly relevant to diagnostic strategies, molecular testing, and access to targeted therapy. However, data from Latin America, particularly from high-incidence subnational regions, remain limited.

To address these gaps, we conducted a comprehensive evaluation of HER2/*ERBB2* abnormalities and sequence variants in 213 additional genes in a cohort of patients with GBC mainly from Salta and Jujuy. We assessed HER2 protein expression by IHC and characterized genomic sequence variants by NGS to capture sequence variants. By integrating standardized IHC with broad genomic profiling in a population drawn from a high-incidence area, this study aimed to (i) determine the prevalence and spectrum of HER2/*ERBB2* abnormalities in GBC from northwestern Argentina, (ii) explore their prognostic associations, (iii) analyze their co-occurrence with sequence variants in other genes, and (iv) provide region-specific evidence to inform the potential implementation of HER2-directed strategies in routine clinical practice and clinical trials.

## 2. Materials and Methods

### 2.1. Patient Selection

A retrospective analysis was conducted using data from patients with GBC diagnosed between 2005 and 2015 and included in the Latin American Intergroup of Gastrointestinal Oncology (ILOGI) database. Patients were eligible if they had a histologically confirmed diagnosis of GBC, available formalin-fixed, paraffin-embedded (FFPE) tissue blocks and available survival data. Written informed consent was obtained from living patients. Patients were recruited from the Argentine provinces of Salta, Jujuy, Tucumán, and Córdoba.

### 2.2. Immunohistochemistry

Samples were processed at Laboratorio San Isidro Patología (San Isidro, Buenos Aires, Argentina) using the Ventana BenchMark ULTRA PLUS automated platform (version VSS v14.1). Immunohistochemistry (IHC) was performed using the anti-HER2 (4B5) antibody, while dualcolor silver-enhanced in situ hybridization (SISH) was performed using the INFORM HER2 Dual ISH DNA Probe Cocktail assay. Both assays were independently evaluated by two pathologists. HER2 protein expression was assessed based on staining intensity and the percentage of positively stained tumor cells. HER2 status was determined according to the American Association of Clinical Oncology (ASCO)/College of American Pathologists (CAP) guidelines for gastric cancer, applying the established scoring criteria for IHC and ISH, which are currently recommended for biliary tract tumors [12].

### 2.3. Next-Generation Sequencing (NGS)

Hematoxylin and eosin (H&E) staining was performed on FFPE tissue sections using standard protocols. Whole-slide images were acquired using a Leica automated imaging system, and tumor areas were annotated by an experienced pathologist for subsequent analysis. Tumor areas were microdissected from 2 to 8 unstained 10 µm FFPE sections using the corresponding H&E slide as a guide. Tissue was deparaffinized with xylene, washed twice with absolute ethanol, and air-dried prior to nucleic acid extraction. DNA was extracted from FFPE tissue, and libraries were prepared using the Agilent SureSelect XT Low Input kit (Agilent Technologies, Santa Clara, CA, USA) Target enrichment was performed using a custom panel covering 214 cancer-related genes and chromosomal regions (Table S1), and paired-end (2 × 150 bp) sequencing was carried out on the Illumina NovaSeq 6000 platform (Illumina, San Diego, CA, USA) following the manufacturer’s protocols. A median coverage depth of 470× was obtained. Sequencing reads were aligned to the GRCh/hg38 reference genome using BWA-MEM, and somatic variants were identified using MuTect2. Variants were annotated using public genomic databases and filtered based on sequencing quality, read depth (≥10 reads supporting the alternative allele), variant allele frequency (≥5% of variant allele frequency for the alternative allele), and population allele frequency to exclude likely germline polymorphisms and false-positive calls. High-impact variants, hotspot missense variants and protein-altering sequence variants, as annotated using the Clinical Annotation of Variants (CAVA) tool (CAVAv.1.3.0)were retained for downstream analyses after manual review using the Integrative Genomics Viewer (IGV). The analyzed variants were classified according to the Association for Molecular Pathology (AMP)/ASCO)/CAP recommendations and the American College of Medical Genetics and Genomics (ACMG) guidelines.

### 2.4. Assessment of HER2/ERBB2 Status

Cases were classified as HER/*ERBB2*-abnormal if they exhibited an IHC score of 3+, and IHC score of 2+ with positive SISH (HER2/CEN17 ratio ≥ 2.00), or *ERBB2* sequence variant detected by NGS.

### 2.5. Statistical Analysis

Co-occurrence and mutual exclusivity of sequence variants were assessed using Fisher’s exact test. Multiple-testing adjustment was performed using the Benjamini–Hochberg method. For each gene pair, a 2 × 2 contingency table was constructed based on the presence or absence of sequence variants and odds ratios (ORs) were calculated. Associations were visualized using a heatmap of log_2_-transformed odds ratios with hierarchical clustering performed using Euclidean distance and Ward’s linkage method. OS was estimated using the Kaplan–Meier method, and survival curves were compared using the log-rank test. Median overall survival (mOS) and corresponding 95% confidence intervals (95% CI) were estimated using the Kaplan–Meier models. Univariate Cox proportional hazards regression models were fitted to estimate hazard ratios (HRs) with corresponding 95% confidence intervals and associated *p*-values. A Firth-penalized Cox regression model was also fitted to reduce small-sample bias. All statistical analyses were performed using R software within the RStudio environment (version 4.4.3; released 28 February 2025). Statistical significance was defined as a two-sided *p*-value of <0.05.

### 2.6. Ethical Considerations

The study was conducted in accordance with the Declaration of Helsinki and was approved (Protocol 17-003417 version 3) by the relevant Institutional Review Boards and Ethics Committees. All living patients provided written informed consent for the use of their tumor tissue and clinical data in this study. Formalin-fixed, paraffin-embedded tissue blocks were de-identified and assigned unique codes to protect patient confidentiality.

## 3. Results

### 3.1. Patients Characteristics

Forty-one tissue blocks from patients with GBC were initially evaluated for IHC and NGS. Eleven were excluded due to low-quality DNA (mean fragment peak length ≤ 500 pb), or insufficient sequencing coverage. Consequently, 30 patients were included in the analysis. The clinical characteristics are presented in Table 1. Of these, 25 patients (83%) were female. Twenty-one tumors (70%) were well or moderately differentiated, and six (20%) were poorly differentiated or undifferentiated. At diagnosis, most patients had advanced disease; 16 patients (53%) had stage IV disease and 8 (27%) had stage III disease. Gallstones were present in 19 patients (63%), absent in 4 (13%), and their status was unknown in 7 (23%).

### 3.2. HER2/ERBB2 Status

HER2/*ERBB2* status, as determined by IHC and NGS, is summarized in Table 2. Detailed descriptions of *ERBB2* variants are provided in Table S2. One tumor could not be evaluated by IHC due to insufficient tumor cellularity. HER2 protein overexpression (IHC 3+) was detected in five patients (17%; 95% CI, 6–35%). Both tumors with an IHC score of 2+ were negative by SISH (Table S3). *ERBB2* variants were identified by NGS in three tumors, involving exons 6 and 21 (10%; 95% CI, 2–27%). Overall, HER2/*ERBB2* abnormalities, defined as HER2 overexpression by IHC and/or *ERBB2* variants detected by NGS, were identified in 8 of 30 patients (27%; 95% CI, 12–46%). None of the tumors harboring *ERBB2* variants exhibited HER2 protein overexpression. Conversely, no HER2-overexpressing tumor harbored an *ERBB2* variant.

### 3.3. Overall Survival Analysis

Given the imbalance in the distribution of early-stage disease (stages I–II) between HER2/*ERBB2* abnormal (1) and normal (5) patients, OS was analyzed exclusively in patients with advanced disease (stages III–IV). Because of the limited sample size and the risk of overfitting associated with multivariable models incorporating multiple covariates, including treatment received, we initially used univariate Cox regression to obtain an unadjusted estimate. When patients were grouped according to combined HER2/*ERBB2* status (IHC and NGS), no statistically significant difference in OS was observed between patients with HER2/*ERBB2*-normal tumors and those with HER2/*ERBB2*-abnormal tumors (HR = 0.40; 95% CI 0.11–1.45) (Figure 1A). Similarly, when OS was analyzed based solely on HER2 protein expression by IHC, no significant difference in survival was detected between patients with HER2-normal and HER2-overexpressing tumors (HR = 0.37; 95% CI 0.73–15.13) (Figure 1B).

To assess the strength of the unadjusted findings, we performed three additional analyses accounting for disease stage: a Cox model stratified by stage (III versus IV) (Figure S1), a stage-stratified log-rank test (Table S4), and a Firth-penalized Cox model adjusted for stage (Table S5). The results were consistent with the unadjusted estimate (HR = 0.40; 95% CI, 0.11–1.45; *p* = 0.16), with no statistically significant association between HER2/*ERBB2* status and OS using the stage-stratified Cox model (HR = 0.46; 95% CI, 0.13–1.70; *p* = 0.24), the stage-stratified log-rank test (*p* = 0.21), or the Firth-penalized Cox model (HR = 0.47; 95% CI, 0.12–1.43; *p* = 0.19). In the stage-specific survival analyses (Figure S1), mOS was numerically longer in patients with HER2/*ERBB2*-abnormal tumors than in those with HER2/*ERBB2*-normal tumors in both stage III (19.5 versus 8.0 months) and stage IV (49.5 versus 12.0 months). These estimates should be interpreted cautiously given the small number of patients within each stratum. Although the analyses accounted for stage, the limited sample size precluded additional adjustment for treatment; therefore, residual confounding by treatment differences cannot be excluded.

### 3.4. Co-Occurrence with Other Molecular Abnormalities

Heatmap analysis (Figure 2) revealed a predominance of positive log_2_ odds ratios, suggesting a potential pattern of co-occurrence among sequence variants across the analyzed gene set. No statistically significant negative associations were identified; however, the limited sample size precludes definitive conclusions regarding mutual exclusivity.

Of the 214 genes analyzed, 179 harbored sequence variants. Exploratory co-occurrence analysis (Table 3) identified nominally significant associations between HER2/*ERBB2* abnormalities and sequence variants in 16 of these 179 genes, using Fisher’s exact test. However, none of these associations remained statistically significant after correction for multiple comparisons using the Benjamini–Hochberg method. Among the 16 genes were *ARID1A* and *SMARCA1* (chromatin remodeling), *EGFR* (involved in the MAPK and PI3K/AKT/mTOR signaling pathways), *KDR* (encoding VEGFR2, a key mediator of angiogenesis), *SETD2* and *KMT2B* (histone methyltransferases), *NCOR1* (nuclear receptor corepressor), *NOTCH2* (cell signaling and development), and *STK11* (also known as *LKB1*, a tumor suppressor gene associated with Peutz–Jeghers syndrome). The distribution of sequence variants across individual tumor samples is presented in Table S6.

## 4. Discussion

In our cohort of 30 Argentine patients with GBC, HER2/*ERBB2* abnormalities were identified in 8 cases (27%). No statistically significant difference in OS was observed between patients with and without HER2/*ERBB2* abnormalities. Exploratory analysis suggested potential co-occurrence of HER2/*ERBB2* alterations with sequence variants in 16 genes.

The clinical characteristics of our study population were similar to those previously reported in patients with GBC [13] (Table 1). The majority of patients were female in their sixth decade of life, with moderately differentiated adenocarcinomas and advanced disease at presentation. Gallstone disease was less frequent in our population; however, this could be related to the incomplete documentation in some medical records. Overall, our study population exhibited clinical characteristics broadly consistent with those reported in other GBC cohorts [13].

We identified HER2/*ERBB2* abnormalities in 27% of patients. None of the three samples harboring *ERBB2* variants had documented HER2 protein overexpression: two had an IHC score of 0, and one could not be evaluated by IHC. Conversely, no *ERBB2* variants were detected in tumors with HER2 overexpression. Certain activating *ERBB2* mutations have been reported to predict response to anti-HER2 therapy [14,15,16]. Excluding the truncating variant c.3457C>T (p.Arg1153Ter), approximately one in four patients in our series might potentially be candidates for HER2-targeted therapy, provided that the remaining variants are confirmed to be oncogenic and predictive of treatment benefit.

Our report is among the first studies in Latin America to evaluate both HER2 protein expression and *ERBB2* gene status in GBC. Roa et al. [8] analyzed the HER2 protein expression in 187 GBC samples from Chile, a country geographically adjacent to the western Argentine provinces represented in our cohort. They identified HER2 overexpression in 24 samples (13%). Our IHC results revealed a comparable prevalence (17%). The frequencies of HER2 protein overexpression reported by Roa et al. and observed in our study are consistent with those reported outside of Latin America [17,18,19,20,21,22] (9–46%). Mondaca et al. [23] analyzed *ERBB2* alterations using NGS in GBC cases from Chile and the USA (Memorial Sloan Kettering Cancer Center, New York). They identified *ERBB2* abnormalities (amplifications, mutations or fusions) in 2/23 (9%) Chilean samples and 33/237 (14%) US samples. Our NGS analysis yielded a comparable frequency (3/30, 10%), although our analysis was limited to sequence variants. Thus, the frequency of HER/*ERBB2* abnormalities observed in our cohort appears broadly consistent with previous reports from Latin America and other regions, although differences in testing methods limit direct comparisons.

In our cohort of patients with stage III–IV GBC, no statistically significant difference in OS was observed between patients with and without HER2/*ERBB2* abnormalities, and this finding remained consistent when the analysis was restricted to HER protein expression. However, the prognostic value of HER2 overexpression in GBC remains controversial. Among studies restricted to patients with GBC, two reported poorer outcomes associated with Her2 positivity [22,24], whereas two others reported longer survival [20,23]. Kumari et al. [20] evaluated 104 GBC cases from India and reported a median OS of 30 months in HER2-overexpressing tumors versus 12 months in HER2-negative tumors. Similarly, Mondaca et al. [23] reported a median OS of 22.3 versus 11.8 months in Chilean patients with abnormal versus normal *ERBB2* status, respectively. In our Argentine cohort, we observed no significant difference in OS according to HER2 status. Notably, studies reporting no adverse prognostic impact of HER2 abnormalities in GBC, including ours, have studied populations from South America and India, regions with high GBC incidence and mortality [10,25]. The observed survival patterns are unlikely to be explained solely by anti-HER2 therapy. as only 27% of the patients received anti-HER2 agents in the Chilean study, and none of the patients in our series or the Indian cohort were treated with HER2-targeted therapy. An alternative hypothesis warranting investigation involves co-occurring genomic alterations. In non-small cell lung cancer (NSCLC), *EGFR* mutations [26] and alterations in the *SMARCA* gene family [27] have been associated with greater sensitivity to chemotherapy and improved clinical outcomes. In our cohort, 64% of patients with HER2/*ERBB2* abnormalities responded to chemotherapy. If the potential co-occurrence of HER2/*ERBB2* abnormalities with *EGFR* and *SMARCA1* variants is confirmed in larger cohorts, further studies could investigate whether these alterations influence chemotherapy responsiveness and survival. Nevertheless, given the limited sample size of our study, the possibility that these observations occurred by chance cannot be excluded.

If the sequence variants identified in *ARID1A, KMT2B,* and *STK11* (*LKB1*) are confirmed to alter protein function, these findings could provide a biological rationale for investigating potential synergistic effects between anti-HER2 therapies and agents targeting the PI3K/AKT/mTOR pathway. First, Berns et al. [28] demonstrated that *ARID1A* loss or mutation is associated with increased Annexin A1 expression and sustained activation of the PI3K/AKT/mTOR pathway, resulting in reduced sensitivity to HER2-targeted therapies. Second, gain-of-function alterations in *KMT2B* may potentially influence sensitivity to PI3K/AKT/mTOR inhibition. Supporting the biological relevance of this pathway, Wang et al. [29] reported that, under conditions of nutrient stress, *KMT2B* promotes mTORC1 signaling and cell survival through methylation of MTR4, a regulator of amino acid transport. Finally, loss of *STK11* function has been shown to accelerate HER2/*ERBB2*-driven mammary tumorigenesis and induce hyperactivation of both mTORC1 and mTORC2 signaling, as evidenced by increased phosphorylation of S6 and AKT [30]. Although these co-occurrence analyses were not statistically significant after correction for multiple testing, the observed patterns deserve further investigation. If confirmed in larger cohorts, the association of HER2/*ERBB2* abnormalities with protein-altering variants in *ARID1A, KMT2B*, and *STK11* could support further evaluation of the combination of anti-HER2 therapies and PI3K/AKT/mTOR pathway inhibitors in both preclinical models and clinical trials involving patients with HER2/*ERBB2*-abnormal GBC.

Similarly, the potential co-occurrence of HER2/*ERBB2* abnormalities with *KDR* (*VEGFR2*) variants, if these variants are confirmed to affect protein function, may suggest a potential role for VEGF-mediated signaling in HER2/*ERBB2*-abnormal tumors. If validated in larger studies, these findings could support preclinical investigation of combined anti-HER2 and VEGF pathway inhibition to determine whether this strategy produces synergistic antitumor activity before its potential evaluation in clinical trials.

Among the 179 genes harboring sequence variants, including genes frequently mutated in biliary tract cancers, we found no statistically significant evidence of mutual exclusivity between HER2/*ERBB2* abnormalities and variants in any of the genes analyzed. However, the limited sample size reduces the statistical power to detect such associations, and these findings require validation in larger cohorts.

The main limitation of our study is its relatively small sample size, which results in wide confidence intervals and increases the risk of both type I and type II errors. Therefore, our findings should be considered hypothesis-generating. We plan to validate these results in a substantially larger cohort. In addition, NGS has limited sensitivity for detecting *ERBB2* amplification, precluding a direct comparison between NGS-based amplification status and HER2 protein assessed by IHC. We followed established guidelines, classifying only IHC 3+ cases and IHC 2+/SISH-positive cases as HER2 positive. Finally, because of the retrospective study design and the absence of a central review of disease progression, the accurate assessment of progression-free survival was considerably limited.

## 5. Conclusions

HER2/*ERBB2* abnormalities, including HER2 protein overexpression and *ERBB2* sequence variants, were frequently identified in Argentine patients with GBC. These abnormalities were not significantly associated with poorer OS and showed potential co-occurrence with variants in genes involved in angiogenesis (*KDR/VEGFR2*), chemotherapy responsiveness (*EGFR* and *SMARCA1*), and regulation of the PI3K/AKT/mTOR signaling pathway (*ARID1A, KMT2B*, and *STK11*). Although none of these associations remained statistically significant after correction for multiple comparisons, they warrant further investigation. If confirmed in larger cohorts, these findings could provide a rationale for functional and clinical studies to clarify the prognostic significance of HER2/*ERBB2* abnormalities, investigate whether concurrent *EGFR* and *SMARCA1* variants influence chemotherapy sensitivity, and explore the potential therapeutic synergy between anti-HER2 agents and inhibitors of the PI3K/AKT/mTOR or VEGF signaling pathways.

## Figures and Tables

**Figure 1:**
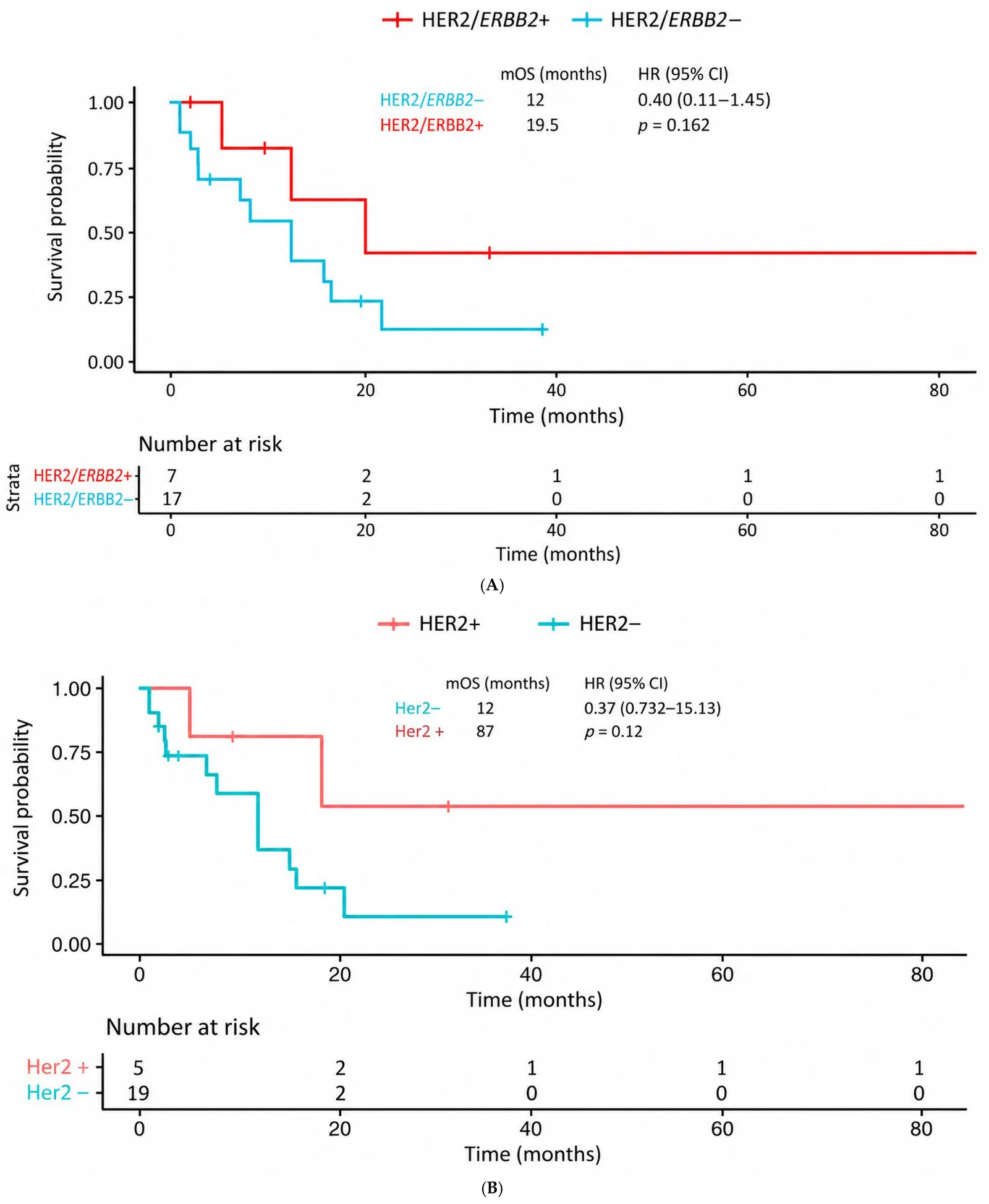
Overall Survival of Stage III-IV GBC patients. (**A**) HER2/*ERBB2* (IHC and NGS) normal (−) vs. abnormal (+); (**B**) HER2 (IHC) normal (−) vs. abnormal (+). mOS = median overall survival; HR = hazard ratio.

**Figure 2:**
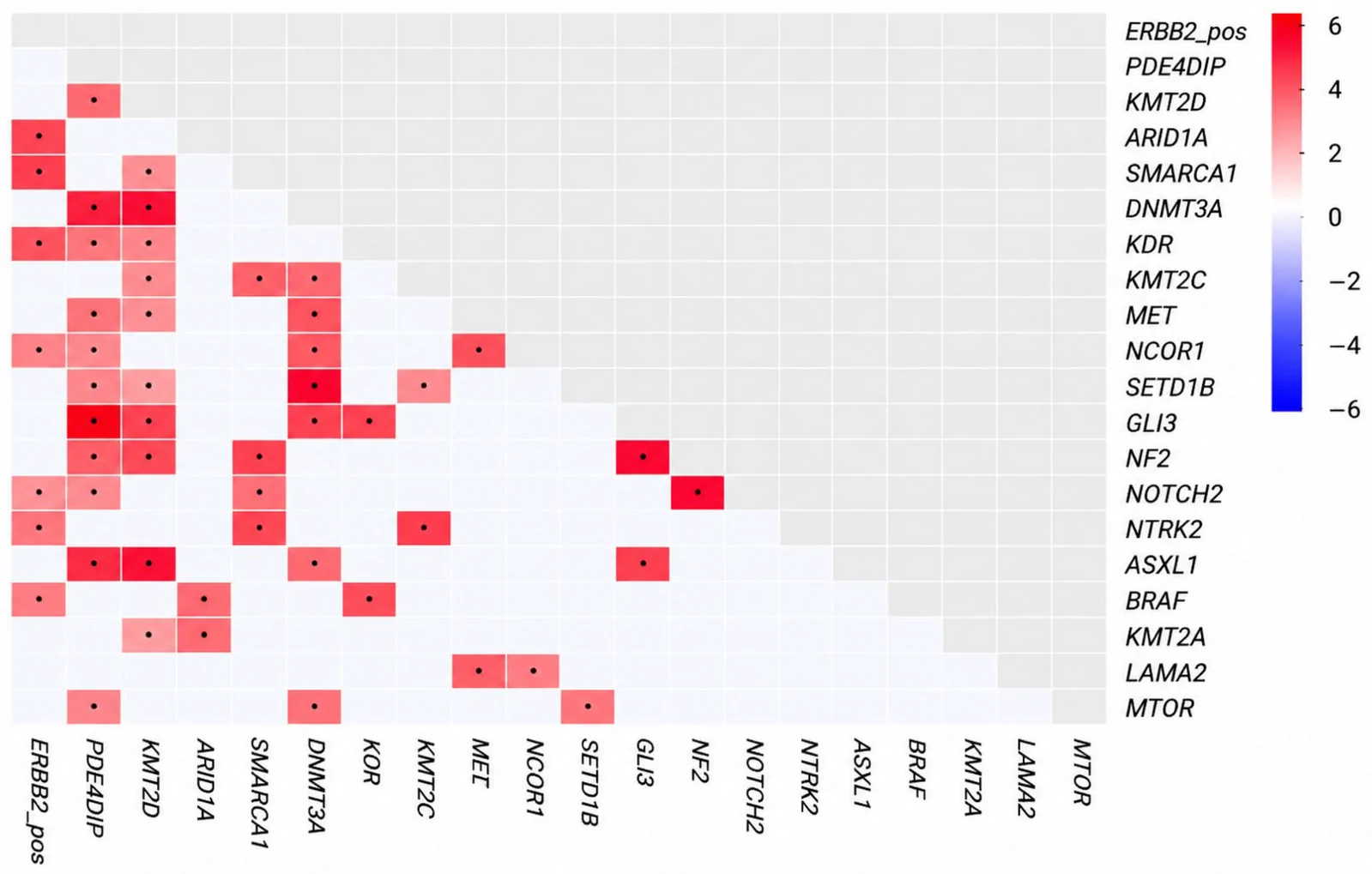
Heatmap of co-occurring gene sequence variants. Statistically significant pairwise co-occurrences are shown in red, whereas nonsignificant co-occurrences are shown in gray.

**Table 1:** Clinical characteristics of patients.

Number	30 (100%)
Age	
Mean	59
Range	37–78
Gender	
Male	5 (17%)
Female	25 (83%)
Histologic type	
Adenocarcinoma	24 (80%)
Squamous cell carcinoma	1 (3%)
Adenosquamous carcinoma	3 (10%)
Carcinoma	1 (3%)
Papillary	1 (3%)
Grade of differentiation	
Well, moderately	21 (70%)
Poorly, undifferentiated	6 (20%)
Unknown	3 (10%)
Stage	
I	4 (13%)
II	1 (3%)
III	8 (27%)
IV	16 (53%)
Unknown	1 (3%)
Lithiasis	
Yes	19 (63%)
No	4 (13%)
Unknown	7 (23%)
Chemotherapy	
Yes	21 (70%)
No	8 (27%)
Unknown	1 (3%)
AntiHER2 therapy	
No	30 (100%)

**Table 2:** HER2/*ERBB2* results.

Sample	IHC Score	NGS Variant
1	0	
2	0	
3	0	
4	0	
5	0	
6	0	
7	3	
8	0	
9	N/A	Exon 21 (c.3457C>T_p.Arg1153Ter)#—VUS
10	0	
11	0	
12	2 (SISH-negative)	
13	0	
14	0	
15	0	
16	0	
17	0	
18	0	
19	3	
20	0	Exon 6 (c.1495C>T_p.Arg499Trp)—VUS
21	0	
22	0	Exon 21 (c.3436C>T_p.Arg1146Trp)—VUS
23	0	
24	3	
25	0	
26	0	
27	3	
28	2 (SISH-negative)	
29	0	
30	3	
HER2/*ERBB2*-abnormal: 8 (27%); 95% CI, 12–46%		

N/A = not available; VUS = variant of uncertain significance; # this variant produces a truncated HER2 protein.

**Table 3:** Exploratory analysis of sequence variants in HER2/*ERBB2*-abnormal versus HER2/*ERBB2*-normal tumours (genes with nominal *p* < 0.11).

Gene	HER2/*ERBB2*-Abnormal (n = 8)	HER2/*ERBB2*-Normal (n = 22)	OR (95% CI)	*p*	q (BH)
*ARID1A*	5/8 (62%)	2/22 (9%)	14.4 (1.57–220)	0.007	0.20
*EGFR*	4/8 (50%)	1/22 (5%)	18.1 (1.37–>1000)	0.011	0.20
*KDR*	4/8 (50%)	1/22 (5%)	18.1 (1.37–>1000)	0.011	0.20
*SMARCA1*	4/8 (50%)	1/22 (5%)	18.1 (1.37–>1000)	0.011	0.20
*SETD2*	4/8 (50%)	2/22 (9%)	9.0 (0.94–133)	0.029	0.21
*NCOR1*	5/8 (62%)	4/22 (18%)	6.9 (0.92–65.8)	0.032	0.21
*NOTCH2*	5/8 (62%)	4/22 (18%)	6.9 (0.92–65.8)	0.032	0.21
*KMT2B*	6/8 (75%)	6/22 (27%)	7.4 (0.98–94.7)	0.034	0.21
*ARID2*	3/8 (38%)	1/22 (5%)	11.2 (0.73–692)	0.048	0.21
*BRAF*	3/8 (38%)	1/22 (5%)	11.2 (0.73–692)	0.048	0.21
*NTRK2*	3/8 (38%)	1/22 (5%)	11.2 (0.73–692)	0.048	0.21
*PDPR*	3/8 (38%)	1/22 (5%)	11.2 (0.73–692)	0.048	0.21
*RADIL*	3/8 (38%)	1/22 (5%)	11.2 (0.73–692)	0.048	0.21
*SF3B1*	3/8 (38%)	1/22 (5%)	11.2 (0.73–692)	0.048	0.21
*SLX4*	3/8 (38%)	1/22 (5%)	11.2 (0.73–692)	0.048	0.21
*STK11*	3/8 (38%)	1/22 (5%)	11.2 (0.73–692)	0.048	0.21
*LAMA2*	4/8 (50%)	3/22 (14%)	5.9 (0.70–58.6)	0.060	0.23
*XIRP2*	4/8 (50%)	3/22 (14%)	5.9 (0.70–58.6)	0.060	0.23
*MET*	3/8 (38%)	2/22 (9%)	5.6 (0.50–84.0)	0.102	0.33
*KMT2A*	5/8 (62%)	6/22 (27%)	4.2 (0.60–36.2)	0.104	0.33
*KMT2C*	5/8 (62%)	6/22 (27%)	4.2 (0.60–36.2)	0.104	0.33
*KMT2D*	5/8 (62%)	6/22 (27%)	4.2 (0.60–36.2)	0.104	0.33

OR = conditional maximum-likelihood odds ratio with exact 95% confidence interval (Fisher’s exact test); q = *p*-value adjusted by the Benjamini–Hochberg method across the 70 genes mutated in ≥4 tumours.

## Data Availability

The original contributions presented in this study are included in the article/Supplementary Material. Further inquiries can be directed to the corresponding authors.

## Supplementary Materials

The following supporting information can be downloaded at: https://www.mdpi.com/article/10.3390/cancers18213461/s1, Figure S1: Overall survival by stage; Table S1: Cancer-Related Genes and Chromosomal Regions Included in the Analysis; Table S2: *ERBB2* variants; Table S3: SISH results; Table S4: Overall Survival Kaplan-Meier Estimates by Stage; Table S5: Overall Survival Hazard Ratios (HER2/*ERBB2*-abnormal vs. normal); Table S6: Clinical characteristics, gene sequencing and Her2 immunohistochemistry results for each patient.
