# Supplementary material for: HER2/ERBB2 and Genomic Alterations in Patients with Gallbladder Cancer from Argentina
Source: Cancers (Basel). 2026 Oct 27;18(21):3461. doi: 10.3390/cancers18213461 (PMC13761896; doi:10.3390/cancers18213461)
Supplement: Supplementary file 1 [file cancers-18-03461-s001.zip › cancers-4629271-supplementary.pdf]

# Supplementary Appendix

## Figure S1. Overall Survival by Stage

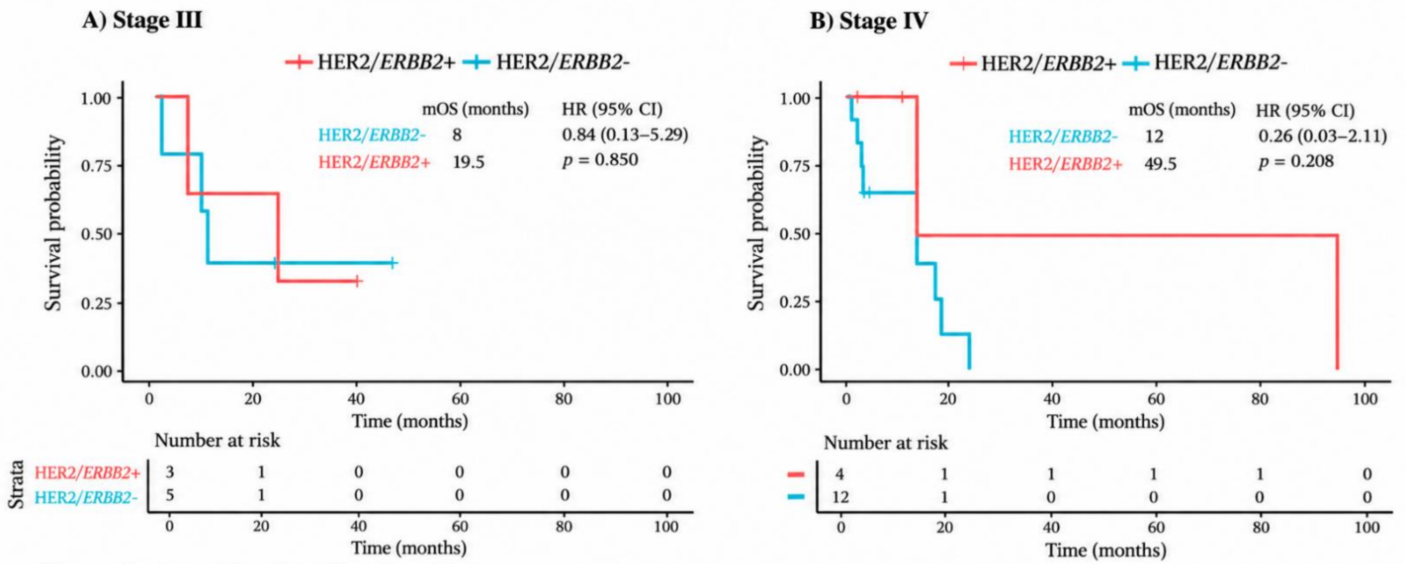


mOS = median Overall Survival; HR = Hazard Ratio.

**Table S1. Cancer-Related Genes and Chromosomal Regions Included in the Analysis.**

| | | | |
|---|---|---|---|
| *PARK2* | *INPP4B* | *USP1* | *GNAS* |
| *ERBB4* | *MDC1* | *SMARCA1* | *FOXP1* |
| *CDK14* | *MLH1* | *KDM3A* | *ATG7* |
| *ASCC3* | *MLH3* | *LAMTOR3* | *MLLT4* |
| *AK5* | *KMT2D* | *VGLL4* | *CDKN2B* |
| *CDK6* | *MLL3* | *TGFBR2* | *SFMBT1* |
| *SH3GL2* | *MORF4L1* | *ARID1A* | *GATA6* |
| *EGFR* | *MRE11A* | *BRCA2* | *CCND3* |
| *ARID2* | *MSH2* | *IDO2* | *FGFR4* |
| *NOTCH2* | *MSH3* | *BLK* | *IKBKB* |
| *FAT1* | *MSH6* | *PIK3R6* | *DDR2* |
| *PBRM1* | *MTOR* | *MTAP* | *CDK12* |
| *MET* | *NBN* | *AKT2* | *SF3B1* |
| *MAP2K4* | *NF1* | *SMAD4* | *MRAS* |
| *MAP4K5* | *NF2* | *RET* | *KMT2A* |
| *FRS2* | *NPEPPS* | *KDR* | *PIK3R1* |
| *AKT1* | *NRAS* | *KRAS* | *TGFBR1* |
| *AKT3* | *PALB2* | *RGS10* | *RNF43* |
| *ALK* | *PCNA* | *MDM4* | *ROBO2* |
| *APC* | *PDPR* | *CTNNB1* | *KMT2B* |
| *ARAF* | *PIK3C2A* | *MDM2* | *NQO1* |
| *ASXL1* | *PLK1* | *SAV1* | *EPHA2* |
| *ATF1* | *PMS1* | *PIK3R5* | *NDC80* |
| *ATR* | *PMS2* | *CCND2* | *PEG3* |
| *ATRIP* | *POLK* | *BIRC2* | *XIRP2* |
| *BAP1* | *POU2F1* | *SBK1* | *LAMA2* |
| *BARD1* | *PTCH1* | *ERBB2* | *RADIL* |
| *BLM* | *RAD18* | *ERBB3* | *UPF3A* |
| *BRAF* | *RAD51* | *BIRC3* | *RPL22* |
| *BRCC3* | *RAD51C* | *WEE1* | *PDE4DIP* |
| *BRIP1* | *RAF1* | *IDO1* | *NBPF1* |
| *CHEK1* | *RBBP8* | *ERRFI1* | *CTBP2* |
| *CHEK2* | *REV1* | *CCND1* | *BAX* |
| *CREBBP* | *REV3L* | *CCNE1* | *CYP4F2* |
| *DDIT4* | *RFC1* | *GLI1* | *AQP7* |
| *DEPTOR* | *RHEB* | *FGF23* | *APOBEC1* |
| *DNMT3A* | *RICTOR* | *FGF3* | *FAM8A1* |
| *EP300* | *RPA1* | *CDKN2A* | *GLI3* |
| *FAM175A* | *RPTOR* | *FGF19* | *GLTSCR1* |
| *FAN1* | *SETD1B* | *CDK2* | *ECT2* |
| *FANCA* | *SETD2* | *MYC* | *WNT5B* |
| *FANCB* | *SLX4* | *MCL1* | *YEATS4* |
| *FANCC* | *SMARCB1* | *CDK4* | *HSF1* |
| *FANCD2* | *SMO* | *FGF4* | *GNA12* |
| *FANCE* | *STAT1* | *ATM* | chr10:123237844-123357972 |
| *FANCF* | *STK11* | *NCOR1* | chr8:32405728-32622558 |
| *FANCG* | *TET1* | *PTEN* | chr4:1795039-1810599 |
| *FANCI* | *TET2* | *TP53* | chr6:117609530-117747018 |
| *FANCL* | *TET3* | *BRCA1* | chr1:156830671-156851642 |
| *FANCM* | *TPTE2* | *RB1* | chr9:87283466-87638505 |
| *FBXW7* | *TSC1* | *ME2* | chr15:88419988-88799962 |
| *HMGB1* | *TSC2* | *PTPN11* | chr5:1293651-1295813 |
| *IDH1* | *UBE2T* | *ACVR2A* | |
| *IDH2* | *UIMC1* | *PIK3CA* | |

## Table S2. *ERBB2* variants

| Sample | Codon | cDNA | Amino acid | VAF | dbSNP | COSMIC | Predicted consequence | Classification | ACMG criteria | Variant effect | Tumor burden (mut/Mb) | Mutational signatures (SBS) contribution (%) |
|---|---|---|---|---|---|---|---|---|---|---|---|---|
| **9** | 1153 | c.3457C>T | p.Arg1153Ter | 0.32 | No | Yes | Truncating (nonsense) | Pathogenic | PVS1, PM2, PM4, PP3 | Loss of function | 1.38 | SBS1 (41.2) |
| **20** | 499 | c.1495C>T | p.Arg499Trp | 0.06 | No | Yes | Missense (GOF) | VUS/Likely pathogenic | PM1, PM2, PP2, PP3 | Gain of function | 0.62 | SBS5 (72.4) |
| **22** | 1146 | c.3436C>T | p.Arg1146Trp | 0.12 | No | Yes | Missense (GOF) | VUS | PM2, PM6, PP2, PP3 | Gain of function | 1.91 | SBS1 (50.1) |

ACMG, American College of Medical Genetics and Genomics; cDNA, coding DNA; GOF, gain of function; mut/Mb, mutations per megabase; PM, moderate evidence; PP, supporting evidence; SBS, single base substitution; VAF, variant allele frequency; VUS, variant of uncertain significance.

**Table S3. SISH results**

| sample ID | nuclei quantification | HER2 | CEN17 | HER/CEN17 |
|---|---|---|---|---|
| 9277 | 20 | 48 | 39 | 1.23 |
| 10091 | 20 | 48 | 39 | 1.23 |

## Table S4. Overall Survival Kaplan–Meier Estimates by Stage

| | **HER2/*ERBB2*** | **n** | **Deaths** | **Median OS, months (95% CI)** | **RMST at 36 months (SE)** |
|---|---|---|---|---|---|
| Stage III–IV | Abnormal | 7 | 4 | 19.5 (12.0–NR) | 22.4 (5.4) |
| | Normal | 17 | 12 | 12.0 (7.0–NR) | 12.8 (2.9) |
| Stage III | Abnormal | 3 | 2 | 19.5 (5.0–NR) | — |
| | Normal | 5 | 3 | 8.0 (7.0–NR) | — |
| Stage IV | Abnormal | 4 | 2 | 49.5 (12.0–NR) | — |
| | Normal | 12 | 9 | 12.0 (2.8–NR) | — |

*N = number of patients; OS = Overall Survival; RMST = Restricted Mean Survival Time;*
*SE = Standard Error; NR = not reached*

**Table S5.** Overall Survival Hazard Ratios (HER2/*ERBB2*-abnormal vs normal)

| **Model** | **HR (95% CI)** | *p* |
|---|---|---|
| Unadjusted Cox | 0.40 (0.11–1.45) | 0.162 |
| Cox stratified by stage | 0.46 (0.13–1.70) | 0.245 |
| Firth-penalized Cox adjusted for stage | 0.47 (0.12–1.43) | 0.191 |
| Log-rank test stratified by stage | — | 0.215 |

HR < 1 indicates a lower risk of death in HER2/*ERBB2*-abnormal tumours.

Proportional-hazards assumption: Schoenfeld test $p = 0.75$.

Stratum-specific estimates are based on very few patients and should be interpreted with caution.

**Table S6.** Clinical characteristics, gene sequencing and Her2 immunohistochemistry results for each patient

| | A | B | C | D | E | F | G | H | I | J | K | L | M | N | O | P | Q | R | S | T | U | V | W | X | Y | Z | AA | AB | AC | AD | AE |
|---|---|---|---|---|---|---|---|---|---|---|---|---|---|---|---|---|---|---|---|---|---|---|---|---|---|---|---|---|---|---|---|
| 1 | protocol number | diagnosis | histologic type | grade of differentiation | type of tissue | gender | age at diagn | lithiasis | OS | chemo | ACVR2A | AK5 | AKT1 | AKT2 | ALK | APC | AQP7 | ARAF | ARID1A | ARID2 | ASCC3 | ASXL1 | ATG7 | ATM | ATR | BAP1 | BARD1 | BAX | BIRC2 | BIRC3 | BLK |
| 2 | 9301 | GB | adenosquamous carcinoma | unknown | surgical specimen | M | 40 | NO | 3+ | NA | | | | | | | | | | | | | | | | | | | | | |
| 3 | 9046 | GB | adenocarcinoma | not reported | surgical specimen | F | 39 | YES | 15.33 | YES | | | | | | exons 6 and 16 | exon 4 | | | exon 7 | | | | exon 17 | | exons 5 and 7 | exon 4 | | | | |
| 4 | 9042 | GB | adenocarcinoma | 3 | surgical specimen | F | 66 | YES | 16 | YES | | | | | | | | | | | | | | | | | | | | | |
| 5 | 9040 | GB | adenocarcinoma | 2 | surgical specimen | M | 58 | YES | 2.66 | YES | | | | | | | | | | | | | | | | | | | | | |
| 6 | 9259 | GB | adenocarcinoma | 2 | surgical specimen | F | 47 | NO | 4+ | YES | | | | exon 12 | | exon 6 | | | | | | | | | | | | | | | |
| 7 | 9261 | GB | squamous carcinoma | 2 | surgical specimen | F | 73 | unknown | 30 | YES | | | | | | exon 16 | | exon 11 | | | exon 36 | | | exon 47 | | | | | | | exon 7 |
| 8 | 9266 | GB | adenocarcinoma | 2 | surgical specimen | F | 57 | YES | 87 | YES | | | | | | | | | exon 20 | | exon 39 | | exon 13, 13 | | | exon 12 | exon 4 | | | | |
| 9 | 9267 | GB | carcinoma | 3 | surgical specimen | F | | unknown | 12 | YES | | | | in 5/6 | | exon 16 | | | | | | exon 11 | | eon 14 | | | | | | | |
| 10 | 9268 | GB | adenocarcinoma | 2 | surgical specimen | F | 51 | NO | 12 | YES | | in 6/7 | | | | | | | exons 2, 20 | | exon 14 | exons 10, 13 | exon 15 | | | | | | | | |
| 11 | 9270 | BILIARY | adenocarcinoma | 2 | surgical specimen | F | 54 | YES | 12 | NO | | | | ex 8 | | | | | | | | | | | | | | | | | |
| 12 | 9272 | GB | adenosquamous carcinoma | 3 | percutaneous biopsy | M | 72 | unknown | 2 | NO | | | | | | | | | | | ex37 | | | | ex44 | | | | | | |
| 13 | 9277 | GB | adenocarcinoma | 2 | surgical specimen | F | 78 | unknown | 37,66+ | YES | | | | | | ex1 | | | | | | | | | | | | | | | |
| 14 | 9278 | GB | adenosquamous carcinoma | 3 | surgical specimen | F | 75 | unknown | 7 | NO | | | ex2 | | | exon 16, 16 | | | ex4 | | | | | | | | | | | | |
| 15 | 9282 | GB | adenocarcinoma | 2 | surgical specimen | F | 75 | YES | 1 | NO | | | | | | ex16 | | | | | | | | ex63 | | | | | | | |
| 16 | 9279 | GB | adenocarcinoma | 3 | surgical specimen | F | 73 | unknown | 1 | NO | | | | | | ex16 | | ex4 | | | | | | | | ex7 | | | | | |
| 17 | 9280 | GB | adenocarcinoma | 2 | surgical specimen | F | 70 | YES | 13 | NO | | | | | | | | | | | | exon 13 | | | | | | | | | |
| 18 | 9286 | GB | papillary carcinoma | | surgical specimen | F | 57 | unknown | 86+ | YES | | | | | | | | | | | | | | | ex22 | | | | | | |
| 19 | 9285 | GB | adenocarcinoma | 2 | surgical specimen | F | 59 | YES | 12 | YES | | | | | | | | | | | | | ex17 | ex10, 31 | | | | | | | |
| 20 | | | | | | | | | | | | | | | | | | | | | | | | | | | | | | | |

| | A | B | C | D | E | F | G | H | I | J | K | L | M | N | O | P | Q | R | S | T | U | V | W | X | Y | Z | AA | AB | AC | AD | AE |
|---|---|---|---|---|---|---|---|---|---|---|---|---|---|---|---|---|---|---|---|---|---|---|---|---|---|---|---|---|---|---|---|
| 1 | protocol number | diagnosis | histologic type | grade of differentiation | type of tissue | gender | age at diagn | lithiasis | OS | chemo | ACVR2A | AK5 | AKT1 | AKT2 | ALK | APC | AQP7 | ARAF | ARID1A | ARID2 | ASCC3 | ASXL1 | ATG7 | ATM | ATR | BAP1 | BARD1 | BAX | BIRC2 | BIRC3 | BLK |
| 2 | 9300 | GB | adenocarcinoma | 3 | surgical specimen | M | 61 | YES | 32+ | YES | | | | | | | | | ex13, exon 20 | ex7 | | ex7 | | | | | | | | | |
| 3 | 9302 | GB | adenocarcinoma | 1 | surgical specimen | F | 61 | NO | 2+ | YES | | | | | | ex16 | | | ex1x2 | ex7 | | | | ex33, ex46 | | ex4 | | | | | |
| 4 | 9304 | GB | adenocarcinoma | 2 | surgical specimen | F | 62 | YES | 4+ | YES | | | | | | | | | | | | | | | | | | | | | |
| 5 | 9287 | GB | adenocarcinoma | 2 | surgical specimen | F | 63 | YES | 49.5 | YES | | | | | | ex16, 16 | | | ex20, ex20 | | | | | | | | | | | | |
| 6 | 9297 | GB | adenocarcinoma | 2 | surgical specimen | F | 54 | YES | 8 | NO | | | | ex9 | | | | | | | | ex13 | | | | | | | | | ex7 |
| 7 | 9295 | GB | adenocarcinoma | 2 | surgical specimen | M | 54 | YES | 9,5+ | YES | | | | ex14 | | | | | | ex8 | | | | | | | | | | | |
| 8 | 9296 | GB | adenocarcinoma | 2 | percutaneous biopsy | F | 37 | YES | 11 | NO | | | | | | | | | | | | | | | | | | | | | |
| 9 | 9299 | GB | adenocarcinoma | 2 | surgical specimen | F | 56 | YES | 19+ | YES | | | | | | | | | | | | | | | ex35 | ex13 | | | | | |
| 10 | 10090 | GB | adenocarcinoma | 2 | surgical specimen | F | 60 | YES | 19.5 | YES | | | | | | ex16 | | | | | | | | ex10 | | ex14 | | | | ex5 | |
| 11 | 10091 | GB | adenocarcinoma | 2 | surgical specimen | F | 62 | YES | 2.8 | YES | | | | | | | | | | | ex13 | | | | | | | | | | |
| 12 | 10092 | GB | adenocarcinoma | 2 | surgical specimen | F | 37 | YES | 21 | YES | | | | | | | | | exon 20 | | | | | | | | | | | | |
| 13 | 10093 | GB | adenocarcinoma | 2 | surgical specimen | F | 46 | YES | 5 | YES | | | | | ex29 | ex16x2 | | | | | | | | | | | | | | | |
| 14 | | | | | | | | | | | | | | | | | | | | | | | | | | | | | | | |
| 15 | VARIANTS | | | | | | | | | | 0 | 1 | 1 | 5 | 1 | 12 | 1 | 2 | 7 | 4 | 5 | 5 | 3 | 7 | 3 | 6 | 2 | 0 | 0 | 1 | 2 |
| 16 | | | | | | | | | | | | | | | | | | | | | | | | | | | | | | | |
| 17 | GB=gallbladder cancer | | | | | | | | | | | | | | | | | | | | | | | | | | | | | | |
| 18 | M=male | | | | | | | | | | | | | | | | | | | | | | | | | | | | | | |
| 19 | F=female | | | | | | | | | | | | | | | | | | | | | | | | | | | | | | |
| 20 | NA=not available | | | | | | | | | | | | | | | | | | | | | | | | | | | | | | |

| | A | B | C | D | E | F | G | H | I | J | K | L | M | N | O | P | Q | R | S | T | U | V | W | X | Y | Z | AA | AB | AC | AD | AE |
|---|---|---|---|---|---|---|---|---|---|---|---|---|---|---|---|---|---|---|---|---|---|---|---|---|---|---|---|---|---|---|---|
| 1 | protocol number | diagnosis | histologic type | grade of differentiation | type of tissue | gender | age at diagn | lithiasis | OS | chemo | BLM | BRAF | BRCA2 | BRIP1 | CCND1 | CCN2 | CCND3 | CCNE1 | CDK2 | CDK4 | CDK6 | CDKN2A | CDK12 | CHEK1 | CHEK2 | CREBBP | CTBP2 | CTNNB1 | CYP4F2 | DDIT4 | DDR2 |
| 2 | 9301 | GB | adenosquamous carcinoma | unknown | surgical specimen | M | 40 | NO | 3+ | NA | | | | | | | ex5 | | | | | | | | | | | | ex12 | | |
| 3 | 9046 | GB | adenocarcinoma | not reported | surgical specimen | F | 39 | YES | 15.33 | YES | exon 5 | | | | | | | | | | | exon 3 | | | | exon 30 | | | | | |
| 4 | 9042 | GB | adenocarcinoma | 3 | surgical specimen | F | 66 | YES | 16 | YES | | | | | | | | | | | | exon 2 | | | | | | | | | |
| 5 | 9040 | GB | adenocarcinoma | 2 | surgical specimen | M | 58 | YES | 2.66 | YES | | exon 16 | | | | | | | | | | exon 2x2 | | | | | | | | | |
| 6 | 9259 | GB | adenocarcinoma | 2 | surgical specimen | F | 47 | NO | 4+ | YES | | | exon 11 | | | | | | | | | exon 2 | | | | | | | | | |
| 7 | 9261 | GB | squamous carcinoma | 2 | surgical specimen | F | 73 | unknown | 30 | YES | | | exon 26 | | | | | | | | | | | | | in 7/8 | | | | | |
| 8 | 9266 | GB | adenocarcinoma | 2 | surgical specimen | F | 57 | YES | 87 | YES | | | | | | | | exon 8 | exons 4, 7 | | exon 4 | | exon 8 | | | exon 23 | exon 5 | | | | |
| 9 | 9267 | GB | carcinoma | 3 | surgical specimen | F | | unknown | 12 | YES | | | | | | | | exon 5 | exon 6 | | | | | | | | in 7/8 | | | | |
| 10 | 9268 | GB | adenocarcinoma | 2 | surgical specimen | F | 51 | NO | 12 | YES | exon 3 | | ex 11 | | | | | | | | | | | | | | | | | | |
| 11 | 9270 | BILIARY | adenocarcinoma | 2 | surgical specimen | F | 54 | YES | 12 | NO | | | | | | | ex 5 | ex 3 | | | | | | | | | | | | | |
| 12 | 9272 | GB | adenosquamous carcinoma | 3 | percutaneous biopsy | M | 72 | unknown | 2 | NO | | | | | | | | | | | | | | | | | | | | | |
| 13 | 9277 | GB | adenocarcinoma | 2 | surgical specimen | F | 78 | unknown | 37,66+ | YES | | | | | | | | | | | | | | | | | | | | | |
| 14 | 9278 | GB | adenosquamous carcinoma | 3 | surgical specimen | F | 75 | unknown | 7 | NO | | | | | | | | | | | | | | | | | | | | | |
| 15 | 9282 | GB | adenocarcinoma | 2 | surgical specimen | F | 75 | YES | 1 | NO | | | | | | | | | | | | | | | | | ex9 | | | | |
| 16 | 9279 | GB | adenocarcinoma | 3 | surgical specimen | F | 73 | unknown | 1 | NO | | | | ex3, ex3 | | | | | | | | | ex 5, 13, 13, 14 | | ex 13 | ex11 | | | | | |
| 17 | 9280 | GB | adenocarcinoma | 2 | surgical specimen | F | 70 | YES | 13 | NO | | | | | | | | | | | | | | | | | | | | | ex16 x 3 |
| 18 | 9286 | GB | papillary carcinoma | | surgical specimen | F | 57 | unknown | 86+ | YES | | | | | | | | | | | | | | | | ex 6, 18 | | | | | ex7 |
| 19 | 9285 | GB | adenocarcinoma | 2 | surgical specimen | F | 59 | YES | 12 | YES | | | | | | | ex5 | | | | | | | | | | | | | | |
| 20 | | | | | | | | | | | | | | | | | | | | | | | | | | | | | | | |

| | A | B | C | D | E | F | G | H | I | J | K | L | M | N | O | P | Q | R | S | T | U | V | W | X | Y | Z | AA | AB | AC | AD | AE |
|---|---|---|---|---|---|---|---|---|---|---|---|---|---|---|---|---|---|---|---|---|---|---|---|---|---|---|---|---|---|---|---|
| 1 | protocol number | diagnosis | histologic type | grade of differentiation | type of tissue | gender | age at diagn | lithiasis | OS | chemo | BLM | BRAF | BRCA2 | BRIP1 | CCND1 | CCN2 | CCND3 | CCNE1 | CDK2 | CDK4 | CDK6 | CDKN2A | CDK12 | CHEK1 | CHEK2 | CREBBP | CTBP2 | CTNNB1 | CYP4F2 | DDIT4 | DDR2 |
| 2 | 9300 | GB | adenocarcinoma | 3 | surgical specimen | M | 61 | YES | 32+ | YES | | ex10 | | | ex4 | | | | | | | | | | | | | | | | ex19 |
| 3 | 9302 | GB | adenocarcinoma | 1 | surgical specimen | F | 61 | NO | 2+ | YES | in 5/6 | ex3,17 | ex25 | ex17 | ex4 | | | ex7 | | | | | | | | ex30x2 | | | ex10 | | |
| 4 | 9304 | GB | adenocarcinoma | 2 | surgical specimen | F | 62 | YES | 4+ | YES | | | | | | | | | | | | | | | | | | | | | |
| 5 | 9287 | GB | adenocarcinoma | 2 | surgical specimen | F | 63 | YES | 49.5 | YES | | in5/6 | | | | | | | | | | | ex10 | | | ex17 | | | | | |
| 6 | 9297 | GB | adenocarcinoma | 2 | surgical specimen | F | 54 | YES | 8 | NO | | | | | | | | | | | | | | | | | | | | | |
| 7 | 9295 | GB | adenocarcinoma | 2 | surgical specimen | M | 54 | YES | 9,5+ | YES | | | | | | | | | | | | | | | | | | | | | |
| 8 | 9296 | GB | adenocarcinoma | 2 | percutaneous biopsy | F | 37 | YES | 11 | NO | | | exon 15 | | | | | | | | | | ex1 | | | | | | | | |
| 9 | 9299 | GB | adenocarcinoma | 2 | surgical specimen | F | 56 | YES | 19+ | YES | | | | | | | | ex8 | | | | | | | | | | | | ex3 | |
| 10 | 10090 | GB | adenocarcinoma | 2 | surgical specimen | F | 60 | YES | 19.5 | YES | | | | | | ex1 | | | | | | | | | | | | ex3 | | | |
| 11 | 10091 | GB | adenocarcinoma | 2 | surgical specimen | F | 62 | YES | 2.8 | YES | | | | | | | | | | | | | | | | | | | | | |
| 12 | 10092 | GB | adenocarcinoma | 2 | surgical specimen | F | 37 | YES | 21 | YES | | | | | | | | | | | | | | | | | | | | | |
| 13 | 10093 | GB | adenocarcinoma | 2 | surgical specimen | F | 46 | YES | 5 | YES | | | | | | | ex5 | | | | | | | | | | | | ex10 | | |
| 14 | | | | | | | | | | | | | | | | | | | | | | | | | | | | | | | |
| 15 | VARIANTS | | | | | | | | | | 3 | 4 | 5 | 2 | 2 | 1 | 4 | 5 | 2 | 0 | 1 | 4 | 4 | 0 | 1 | 7 | 3 | 1 | 3 | 1 | 3 |
| 16 | | | | | | | | | | | | | | | | | | | | | | | | | | | | | | | |
| 17 | GB=gallbladder cancer | | | | | | | | | | | | | | | | | | | | | | | | | | | | | | |
| 18 | M=male | | | | | | | | | | | | | | | | | | | | | | | | | | | | | | |
| 19 | F=female | | | | | | | | | | | | | | | | | | | | | | | | | | | | | | |
| 20 | NA=not available | | | | | | | | | | | | | | | | | | | | | | | | | | | | | | |

| | A | B | C | D | E | F | G | H | I | J | K | L | M | N | O | P | Q | R | S | T | U | V | W | X | Y | Z | AA | AB | AC | AD | AE |
|---|---|---|---|---|---|---|---|---|---|---|---|---|---|---|---|---|---|---|---|---|---|---|---|---|---|---|---|---|---|---|---|
| 1 | protocol number | diagnosis | histologic type | grade of differentiation | type of tissue | gender | age at diagn | lithiasis | OS | chemo | DEPTOR | DNMT3A | ECT2 | EGFR | EPHA2 | EP300 | ERBB2 | ERBB3 | ERBB4 | FAM8A1 | FAM175A | FAN1 | FANCA | FANCC | FANCD2 | FANCE | FANCF | FANCG | FANCI | FANCL | FANCM |
| 2 | 9301 | GB | adenosquamous carcinoma | unknown | surgical specimen | M | 40 | NO | 3+ | NA | | | | | | | | | | | | | | | ex9 | | | | | | |
| 3 | 9046 | GB | adenocarcinoma | not reported | surgical specimen | F | 39 | YES | 15.33 | YES | | | | | | | | | | | | | | | | | | | | | |
| 4 | 9042 | GB | adenocarcinoma | 3 | surgical specimen | F | 66 | YES | 16 | YES | | | | | | | | | | | | | | | | | | | | | |
| 5 | 9040 | GB | adenocarcinoma | 2 | surgical specimen | M | 58 | YES | 2.66 | YES | | | | exon 2 | | | | | | | | | exon 38 | | | | | | | | |
| 6 | 9259 | GB | adenocarcinoma | 2 | surgical specimen | F | 47 | NO | 4+ | YES | | | | | | | | | | | | | | | | | | | | | |
| 7 | 9261 | GB | squamous carcinoma | 2 | surgical specimen | F | 73 | unknown | 30 | YES | | | | | | | | | | | | | | | | | | | | | |
| 8 | 9266 | GB | adenocarcinoma | 2 | surgical specimen | F | 57 | YES | 87 | YES | | | | exon 26 | | exons 4, 14, 30x2 | | | | | | | | | | | | | exon 23 | | |
| 9 | 9267 | GB | carcinoma | 3 | surgical specimen | F | | unknown | 12 | YES | | exon 3 | | | | in 5/6 | | | | exon 3x2 | | | | | | | | | | | |
| 10 | 9268 | GB | adenocarcinoma | 2 | surgical specimen | F | 51 | NO | 12 | YES | | exon 3, 14, 17 | | | | | exon 21 | | | | | exon2 | | exon 9 | | | | exons 12, 14 | | | |
| 11 | 9270 | BILIARY | adenocarcinoma | 2 | surgical specimen | F | 54 | YES | 12 | NO | | | | | | exon 2 | | ex 4 | | | | | | | ex 11 | | | | | | |
| 12 | 9272 | GB | adenosquamous carcinoma | 3 | percutaneous biopsy | M | 72 | unknown | 2 | NO | | | | | | ex11 | | | | | | | | | | | | | | | |
| 13 | 9277 | GB | adenocarcinoma | 2 | surgical specimen | F | 78 | unknown | 37,66+ | YES | | | | | | | | | | | | | | | | | | | | | |
| 14 | 9278 | GB | adenosquamous carcinoma | 3 | surgical specimen | F | 75 | unknown | 7 | NO | | | | | | | | | | ex4 | | | | | ex22 | ex2 | | | | | |
| 15 | 9282 | GB | adenocarcinoma | 2 | surgical specimen | F | 75 | YES | 1 | NO | | | | | | | | | | | | | | | | | | | | | |
| 16 | 9279 | GB | adenocarcinoma | 3 | surgical specimen | F | 73 | unknown | 1 | NO | | ex8 | | | | | | | | | | | | | | | | exon 1 | | | |
| 17 | 9280 | GB | adenocarcinoma | 2 | surgical specimen | F | 70 | YES | 13 | NO | | | | | | | | | ex27 | | | | | | | | | | | | |
| 18 | 9286 | GB | papillary carcinoma | | surgical specimen | F | 57 | unknown | 86+ | YES | | | | | | | | | | | | | | | ex10 | | | | | | |
| 19 | 9285 | GB | adenocarcinoma | 2 | surgical specimen | F | 59 | YES | 12 | YES | | | | | | | | | | | | | | | | | | | ex32 | | |
| 20 | | | | | | | | | | | | | | | | | | | | | | | | | | | | | | | |

| | A | B | C | D | E | F | G | H | I | J | K | L | M | N | O | P | Q | R | S | T | U | V | W | X | Y | Z | AA | AB | AC | AD | AE |
|---|---|---|---|---|---|---|---|---|---|---|---|---|---|---|---|---|---|---|---|---|---|---|---|---|---|---|---|---|---|---|---|
| 1 | protocol number | diagnosis | histologic type | grade of differentiation | type of tissue | gender | age at diagn | lithiasis | OS | chemo | DEPTOR | DNMT3A | ECT2 | EGFR | EPHA2 | EP300 | ERBB2 | ERBB3 | ERBB4 | FAM8A1 | FAM175A | FAN1 | FANCA | FANCC | FANCD2 | FANCE | FANCF | FANCG | FANCI | FANCL | FANCM |
| 2 | 9300 | GB | adenocarcinoma | 3 | surgical specimen | M | 61 | YES | 32+ | YES | | | | ex22 | | | | | | | | | | | | | | | | | ex9 |
| 3 | 9302 | GB | adenocarcinoma | 1 | surgical specimen | F | 61 | NO | 2+ | YES | | ex18 | | | | ex2,31 | ex6 | ex10 | ex25 | | ex5, ex5 | | ex42 | | | ex2, ex8 | | | | | |
| 4 | 9304 | GB | adenocarcinoma | 2 | surgical specimen | F | 62 | YES | 4+ | YES | | | | | | | | | | | | | | | | | | | | | |
| 5 | 9287 | GB | adenocarcinoma | 2 | surgical specimen | F | 63 | YES | 49.5 | YES | | | | | ex3 | ex2 | ex21 | | | | | | | | | | | | ex35 | | |
| 6 | 9297 | GB | adenocarcinoma | 2 | surgical specimen | F | 54 | YES | 8 | NO | | ex12 | | | | | | | | | | | | | | | | | | | |
| 7 | 9295 | GB | adenocarcinoma | 2 | surgical specimen | M | 54 | YES | 9,5+ | YES | | | | ex28 | | | | | | | | | | | | | | | | | |
| 8 | 9296 | GB | adenocarcinoma | 2 | percutaneous biopsy | F | 37 | YES | 11 | NO | | | | | | | | | | | | | ex17 | | | | | ex12 | | | |
| 9 | 9299 | GB | adenocarcinoma | 2 | surgical specimen | F | 56 | YES | 19+ | YES | | | ex22 | | | | | | | | | | | | | | | | | | |
| 10 | 10090 | GB | adenocarcinoma | 2 | surgical specimen | F | 60 | YES | 19.5 | YES | | | | | | | | | | In4/5 | | | | | | | | | | | |
| 11 | 10091 | GB | adenocarcinoma | 2 | surgical specimen | F | 62 | YES | 2.8 | YES | | | | | | | | | | | | | | | | | | | | | |
| 12 | 10092 | GB | adenocarcinoma | 2 | surgical specimen | F | 37 | YES | 21 | YES | | | | | ex2,3 | | | | | | | | | | | | | ex12 | | | |
| 13 | 10093 | GB | adenocarcinoma | 2 | surgical specimen | F | 46 | YES | 5 | YES | | | | ex22 | | | | | ex27 | | | | | | | In 9/10 | | | | | |
| 14 | | | | | | | | | | | | | | | | | | | | | | | | | | | | | | | |
| 15 | VARIANTS | | | | | | | | | | 0 | 5 | 1 | 5 | 2 | 6 | 3 | | | | | | | | | | | | | | |
| 16 | | | | | | | | | | | | | | | | | | | | | | | | | | | | | | | |
| 17 | GB=gallbladder cancer | | | | | | | | | | | | | | | | | | | | | | | | | | | | | | |
| 18 | M=male | | | | | | | | | | | | | | | | | | | | | | | | | | | | | | |
| 19 | F=female | | | | | | | | | | | | | | | | | | | | | | | | | | | | | | |
| 20 | NA=not available | | | | | | | | | | | | | | | | | | | | | | | | | | | | | | |

| | A | B | C | D | E | F | G | H | I | J | K | L | M | N | O | P | Q | R | S | T | U | V | W | X | Y | Z | AA | AB | AC | AD | AE |
|---|---|---|---|---|---|---|---|---|---|---|---|---|---|---|---|---|---|---|---|---|---|---|---|---|---|---|---|---|---|---|---|
| 1 | protocol number | diagnosis | histologic type | grade of differentiation | type of tissue | gender | age at diagn | lithiasis | OS | chemo | FAT1 | FBXW7 | FGFR2 | FGFR3 | FGFR4 | FMT2D | FOXP1 | FRS2 | GATA6 | GLI1 | GLI3 | GLTSCR1 | GNA12 | GNAS | GNAS12 | Her2 | Her2 SISH | HSF1 | IDH1 | IDH2 | IDO2 |
| 2 | 9301 | GB | adenosquamous carcinoma | unknown | surgical specimen | M | 40 | NO | 3+ | NA | | | | | | | | | | | | ex6 | | | | 0 | | | | | |
| 3 | 9046 | GB | adenocarcinoma | not reported | surgical specimen | F | 39 | YES | 15.33 | YES | | | | | | | | | | | | | | | | 0 | | | | | |
| 4 | 9042 | GB | adenocarcinoma | 3 | surgical specimen | F | 66 | YES | 16 | YES | exon 5 | | | | | | | | | | | | | | | 0 | | | | | |
| 5 | 9040 | GB | adenocarcinoma | 2 | surgical specimen | M | 58 | YES | 2.66 | YES | | | | | | | | | | | | exon 6 | | | | 0 | | | | | |
| 6 | 9259 | GB | adenocarcinoma | 2 | surgical specimen | F | 47 | NO | 4+ | YES | exon 5 | | | exon 3 | | | | | | | | | | | | 0 | | | | | |
| 7 | 9261 | GB | squamous carcinoma | 2 | surgical specimen | F | 73 | unknown | 30 | YES | | | | | | | exon 16 | | | | | | | | | 0 | | | | | |
| 8 | 9266 | GB | adenocarcinoma | 2 | surgical specimen | F | 57 | YES | 87 | YES | ex17 | | | | | | exon 19 | | | | | | | | exon 1x3 | 3 | | | exon 7 | | |
| 9 | 9267 | GB | carcinoma | 3 | surgical specimen | F | | unknown | 12 | YES | | | exon 11 | | | | | | | | exon 3 | | | | | 0 | | | | | |
| 10 | 9268 | GB | adenocarcinoma | 2 | surgical specimen | F | 51 | NO | 12 | YES | | | exons 4, 6, 7 | | exon 6 | | | | | exon 7 | | | | | | | | | | in 5/6 | |
| 11 | 9270 | BILIARY | adenocarcinoma | 2 | surgical specimen | F | 54 | YES | 12 | NO | ex 2 | | | | | | | | | | | | | | | 0 | | | | | ex 8 |
| 12 | 9272 | GB | adenosquamous carcinoma | 3 | percutaneous biopsy | M | 72 | unknown | 2 | NO | ex19 | | ex6 | | | | | | | | | | | | | 0 | | In 3/4 | | | |
| 13 | 9277 | GB | adenocarcinoma | 2 | surgical specimen | F | 78 | unknown | 37,66+ | YES | | | | | | | | | | | | | | | | 2 neg | | | | | |
| 14 | 9278 | GB | adenosquamous carcinoma | 3 | surgical specimen | F | 75 | unknown | 7 | NO | ex10 | | | | | | ex13 | | | | | | | | | 0 | | | | | |
| 15 | 9282 | GB | adenocarcinoma | 2 | surgical specimen | F | 75 | YES | 1 | NO | ex25 | | | ex3? | | | | | | | | | | | | 0 | | | | | |
| 16 | 9279 | GB | adenocarcinoma | 3 | surgical specimen | F | 73 | unknown | 1 | NO | ex 10, ex 10, 12 | | | | | | | | | | | | | | | 0 | | | | | |
| 17 | 9280 | GB | adenocarcinoma | 2 | surgical specimen | F | 70 | YES | 13 | NO | ex17 | ex1 | | | | | | | | | | | | | | 0 | | | | | |
| 18 | 9286 | GB | papillary carcinoma | | surgical specimen | F | 57 | unknown | 86+ | YES | ex2 | ex11 | | | | | | | | | | | | | | 0 | | | | | |
| 19 | 9285 | GB | adenocarcinoma | 2 | surgical specimen | F | 59 | YES | 12 | YES | ex2, 25 | | | | | | | | | | | ex15 | | | | 0 | | | | | |
| 20 | | | | | | | | | | | | | | | | | | | | | | | | | | | | | | | |

| | A | B | C | D | E | F | G | H | I | J | K | L | M | N | O | P | Q | R | S | T | U | V | W | X | Y | Z | AA | AB | AC | AD | AE |
|---|---|---|---|---|---|---|---|---|---|---|---|---|---|---|---|---|---|---|---|---|---|---|---|---|---|---|---|---|---|---|---|
| 1 | protocol number | diagnosis | histologic type | grade of differentiation | type of tissue | gender | age at diagn | lithiasis | OS | chemo | FAT1 | FBXW7 | FGFR2 | FGFR3 | FGFR4 | FMT2D | FOXP1 | FRS2 | GATA6 | GLI1 | GLI3 | GLTSCR1 | GNA12 | GNAS | GNAS12 | Her2 | Her2 SISH | HSF1 | IDH1 | IDH2 | IDO2 |
| 2 | 9300 | GB | adenocarcinoma | 3 | surgical specimen | M | 61 | YES | 32+ | YES | ex2 | ex5 | | | | | | | | | ex9, ex3 | ex13 | | | | 3 | | | | | |
| 3 | 9302 | GB | adenocarcinoma | 1 | surgical specimen | F | 61 | NO | 2+ | YES | ex2,8,10,10,19,2 1 | | | | | | ex8 | | | ex7,10 | ex3,10,15 | ex15 | | | | 0 | | | | | ex4,10 |
| 4 | 9304 | GB | adenocarcinoma | 2 | surgical specimen | F | 62 | YES | 4+ | YES | | | ex2 | | | | | | | ex10 | | | | | | 0 | | | | | |
| 5 | 9287 | GB | adenocarcinoma | 2 | surgical specimen | F | 63 | YES | 49.5 | YES | | exon 11 | | | | | | | | | | | | | | 0 | | | | | |
| 6 | 9297 | GB | adenocarcinoma | 2 | surgical specimen | F | 54 | YES | 8 | NO | | | | | | | | | ex6 | | ex 15, 15 | | | | | 0 | | | | | |
| 7 | 9295 | GB | adenocarcinoma | 2 | surgical specimen | M | 54 | YES | 9,5+ | YES | | | ex14 | | | | | | | | | | | | | 3 | | | | | |
| 8 | 9296 | GB | adenocarcinoma | 2 | percutaneous biopsy | F | 37 | YES | 11 | NO | | | | | | | | | | | | | | | | 0 | | ex7 | | | |
| 9 | 9299 | GB | adenocarcinoma | 2 | surgical specimen | F | 56 | YES | 19+ | YES | | | | | | | | | | | | | | | | 0 | | | | | |
| 10 | 10090 | GB | adenocarcinoma | 2 | surgical specimen | F | 60 | YES | 19.5 | YES | | | | | | | | | | | | | | | | 3 | | ex10 | | | |
| 11 | 10091 | GB | adenocarcinoma | 2 | surgical specimen | F | 62 | YES | 2.8 | YES | | | | | | | | | | | | | | | | 2 neg | | | | | |
| 12 | 10092 | GB | adenocarcinoma | 2 | surgical specimen | F | 37 | YES | 21 | YES | | | | | | | | | | ex10 | | | | | | 0 | | | | | |
| 13 | 10093 | GB | adenocarcinoma | 2 | surgical specimen | F | 46 | YES | 5 | YES | ex2 | | | | | | | | | | | | | ex1x2 | | 3 | | | | | |
| 14 | | | | | | | | | | | | | | | | | | | | | | | | | | | | | | | |
| 15 | VARIANTS | | | | | | | | | | | | | | | | | | | | | | | | | | | 3 | 1 | 1 | 2 |
| 16 | | | | | | | | | | | | | | | | | | | | | | | | | | | | | | | |
| 17 | GB=gallbladder cancer | | | | | | | | | | | | | | | | | | | | | | | | | | | | | | |
| 18 | M=male | | | | | | | | | | | | | | | | | | | | | | | | | | | | | | |
| 19 | F=female | | | | | | | | | | | | | | | | | | | | | | | | | | | | | | |
| 20 | NA=not available | | | | | | | | | | | | | | | | | | | | | | | | | | | | | | |

| | A | B | C | D | E | F | G | H | I | J | K | L | M | N | O | P | Q | R | S | T | U | V | W | X | Y | Z | AA | AB | AC | AD | AE |
|---|---|---|---|---|---|---|---|---|---|---|---|---|---|---|---|---|---|---|---|---|---|---|---|---|---|---|---|---|---|---|---|
| 1 | protocol number | diagnosis | histologic type | grade of differentiation | type of tissue | gender | age at diagn | lithiasis | OS | chemo | IKBKB | INPP4B | KDM3A | KDR | KMT2A | KMT2B | KMT2C | KMT2D | KRAS | LAMA2 | MAP4K5 | MCL1 | MDC1 | ME2 | MET | MLH1 | MLH3 | MLLT4 | MORF4L1 | MRAS | MRE11A |
| 2 | 9301 | GB | adenosquamous carcinoma | unknown | surgical specimen | M | 40 | NO | 3+ | NA | | | | | | | | | | | | | | | | | | ex29 | | | |
| 3 | 9046 | GB | adenocarcinoma | not reported | surgical specimen | F | 39 | YES | 15.33 | YES | | | | | | | | | | | | | exon 10 | | | | | exon 29 | | | |
| 4 | 9042 | GB | adenocarcinoma | 3 | surgical specimen | F | 66 | YES | 16 | YES | | | | | | | | | | exon 5 | | | | | | | exon 1 | | | | |
| 5 | 9040 | GB | adenocarcinoma | 2 | surgical specimen | M | 58 | YES | 2.66 | YES | | | | | | | | | exon 2 | | | | | | | | | | | | |
| 6 | 9259 | GB | adenocarcinoma | 2 | surgical specimen | F | 47 | NO | 4+ | YES | | | | | exon 3 | | | | | | | | | | | | | | | | |
| 7 | 9261 | GB | squamous carcinoma | 2 | surgical specimen | F | 73 | unknown | 30 | YES | | | | | | | exon 38 | | | | | | exon 10x3 | | | | | | | | |
| 8 | 9266 | GB | adenocarcinoma | 2 | surgical specimen | F | 57 | YES | 87 | YES | in 20/21 | | | | exon 4, 26, 27 | exon 20, 23 | exons 43, 43, 47,50, 52 | exons 19, 45 | | | | | exon 10 | | | | | | | | |
| 9 | 9267 | GB | carcinoma | 3 | surgical specimen | F | | unknown | 12 | YES | | | | exon 28 | | exon 28x2 | exon 43 | exons 10, 28, 33 | | | | | exon 10 | | | exon 6 | | exon 29 | | | |
| 10 | 9268 | GB | adenocarcinoma | 2 | surgical specimen | F | 51 | NO | 12 | YES | | | | | exons 11, 25 | exon 16 | exons 24, 36, 43 | exon 53 | | exons 24, 26, 32, 58 | | | | | exon 19 | | exons 1, 6 | exon 13 | exon 13 | | |
| 11 | 9270 | BILIARY | adenocarcinoma | 2 | surgical specimen | F | 54 | YES | 12 | NO | | | | | | | | | | ex 6 | | | | ex 2 | | | | | | | |
| 12 | 9272 | GB | adenosquamous carcinoma | 3 | percutaneous biopsy | M | 72 | unknown | 2 | NO | | | | | | | ex14 | | | | | | ex8 | | | | ex11 | | | | |
| 13 | 9277 | GB | adenocarcinoma | 2 | surgical specimen | F | 78 | unknown | 37,66+ | YES | | | | | | | | | | | ex22 | | | | | | | | | | |
| 14 | 9278 | GB | adenosquamous carcinoma | 3 | surgical specimen | F | 75 | unknown | 7 | NO | | | | | ex28 | ex20, ex20 | | | | | | | | | | | | | | | |
| 15 | 9282 | GB | adenocarcinoma | 2 | surgical specimen | F | 75 | YES | 1 | NO | | | | | | ex28 | | | | | | | ex8 | | | | | | | | |
| 16 | 9279 | GB | adenocarcinoma | 3 | surgical specimen | F | 73 | unknown | 1 | NO | | | | | ex3 | ex13 | ex36 | ex12 | | | | | | | | | | | | | ex6 |
| 17 | 9280 | GB | adenocarcinoma | 2 | surgical specimen | F | 70 | YES | 13 | NO | | | | | | | | exon 48 | | | | | | | | | | | | | |
| 18 | 9286 | GB | papillary carcinoma | | surgical specimen | F | 57 | unknown | 86+ | YES | | | | | ex36, ex27 | | ex57 | ex49 | | ex18 | | | | | ex2 | | | | ex11 | | |
| 19 | 9285 | GB | adenocarcinoma | 2 | surgical specimen | F | 59 | YES | 12 | YES | | | | | | | ex15 | | | | | | ex10 | | | | | | | | |
| 20 | | | | | | | | | | | | | | | | | | | | | | | | | | | | | | | |

| | A | B | C | D | E | F | G | H | I | J | K | L | M | N | O | P | Q | R | S | T | U | V | W | X | Y | Z | AA | AB | AC | AD | AE |
|---|---|---|---|---|---|---|---|---|---|---|---|---|---|---|---|---|---|---|---|---|---|---|---|---|---|---|---|---|---|---|---|
| 1 | protocol number | diagnosis | histologic type | grade of differentiation | type of tissue | gender | age at diagn | lithiasis | OS | chemo | IKBKB | INPP4B | KDM3A | KDR | KMT2A | KMT2B | KMT2C | KMT2D | KRAS | LAMA2 | MAP4K5 | MCL1 | MDC1 | ME2 | MET | MLH1 | MLH3 | MLLT4 | MORF4L1 | MRAS | MRE11A |
| 2 | 9300 | GB | adenocarcinoma | 3 | surgical specimen | M | 61 | YES | 32+ | YES | | | | ex21 | ex3 | ex12 | ex8 | ex10, ex34 | | ex 52, 56 | | | | | | | | | | | |
| 3 | 9302 | GB | adenocarcinoma | 1 | surgical specimen | F | 61 | NO | 2+ | YES | | | | ex27 | ex3 | | ex57 | ex39 | | ex7,33 | | | ex10x2,13 | | ex7 | | | | | | |
| 4 | 9304 | GB | adenocarcinoma | 2 | surgical specimen | F | 62 | YES | 4+ | YES | | | ex12 | | | | | ex11 | | | | | | | | | | | | | ex17 |
| 5 | 9287 | GB | adenocarcinoma | 2 | surgical specimen | F | 63 | YES | 49.5 | YES | | | | ex8 | ex27 | | | ex31 | | | | | | | | | | ex29 | | | |
| 6 | 9297 | GB | adenocarcinoma | 2 | surgical specimen | F | 54 | YES | 8 | NO | | | | | | ex27 | | ex34 | | | | | | | ex2 | | | | | | |
| 7 | 9295 | GB | adenocarcinoma | 2 | surgical specimen | M | 54 | YES | 9,5+ | YES | | | | | | ex21 | ex48 | | | | | | ex 8, 10 | | | exon 8 | | | | | ex14 |
| 8 | 9296 | GB | adenocarcinoma | 2 | percutaneous biopsy | F | 37 | YES | 11 | NO | | | | | ex27 | | | | | | | | | | | | | | | | |
| 9 | 9299 | GB | adenocarcinoma | 2 | surgical specimen | F | 56 | YES | 19+ | YES | | | | | ex3, ex27 | | | | | | | | | | | | | | | | |
| 10 | 10090 | GB | adenocarcinoma | 2 | surgical specimen | F | 60 | YES | 19.5 | YES | | | | ex12 | | ex6 | | | | ex53 | | | | | ex15 | | | | | | |
| 11 | 10091 | GB | adenocarcinoma | 2 | surgical specimen | F | 62 | YES | 2.8 | YES | | | | | | | | | | | | | | | | | | | | | |
| 12 | 10092 | GB | adenocarcinoma | 2 | surgical specimen | F | 37 | YES | 21 | YES | | | | | | ex6 | | | | | | ex4,5 | | | | | | | ex8 | | |
| 13 | 10093 | GB | adenocarcinoma | 2 | surgical specimen | F | 46 | YES | 5 | YES | | | | | | ex3,26 | | | | | | | | | | | | | | | ex10 |
| 14 | | | | | | | | | | | | | | | | | | | | | | | | | | | | | | | |
| 15 | VARIANTS | | | | | | | | | | 1 | 0 | 1 | 5 | 11 | 12 | 11 | 11 | 1 | 7 | 1 | 1 | 9 | 1 | 5 | 2 | 3 | 5 | 3 | 0 | 3 |
| 16 | | | | | | | | | | | | | | | | | | | | | | | | | | | | | | | |
| 17 | GB=gallbladder cancer | | | | | | | | | | | | | | | | | | | | | | | | | | | | | | |
| 18 | M=male | | | | | | | | | | | | | | | | | | | | | | | | | | | | | | |
| 19 | F=female | | | | | | | | | | | | | | | | | | | | | | | | | | | | | | |
| 20 | NA=not available | | | | | | | | | | | | | | | | | | | | | | | | | | | | | | |

| # | A | B | C | D | E | F | G | H | I | J | K | L | M | N | O | P | Q | R | S | T | U | V | W | X | Y | Z | AA | AB | AC | AD | AE |
|---|---|---|---|---|---|---|---|---|---|---|---|---|---|---|---|---|---|---|---|---|---|---|---|---|---|---|---|---|---|---|---|
| 1 | protocol number | diagnosis | histologic type | grade of differentiation | type of tissue | gender | age at diagn | lithiasis | OS | chemo | MSH2 | MSH3 | MSH6 | MTOR | MYC | NBN | NBPF1 | NCOR1 | NF1 | NF2 | NOTCH2 | NPEPPS | NRAS | NRG1 | NTRK1 | NTRK2 | NTRK3 | PALB2 | PARK2 | PBRM1 | PCNA |
| 2 | 9301 | GB | adenosquamous carcinoma | unknown | surgical specimen | M | 40 | NO | 3+ | NA | | | | | | | | | | | | | | | | | | | | | |
| 3 | 9046 | GB | adenocarcinoma | not reported | surgical specimen | F | 39 | YES | 15.33 | YES | | | | | | | | | | | | exon 7 | | | | | | | | | |
| 4 | 9042 | GB | adenocarcinoma | 3 | surgical specimen | F | 66 | YES | 16 | YES | | | | | | | | | | | | | | | | | | | | | |
| 5 | 9040 | GB | adenocarcinoma | 2 | surgical specimen | M | 58 | YES | 2.66 | YES | | exon 13 | | | | | | | | | | | | exons 2 and 3 | | | | | | | |
| 6 | 9259 | GB | adenocarcinoma | 2 | surgical specimen | F | 47 | NO | 4+ | YES | | | | | | | | | | | | | | | | | | | | | |
| 7 | 9261 | GB | squamous carcinoma | 2 | surgical specimen | F | 73 | unknown | 30 | YES | | | | | | | | | exon 27 | | | | | | | | | exon 9 | | | |
| 8 | 9266 | GB | adenocarcinoma | 2 | surgical specimen | F | 57 | YES | 87 | YES | | | | exon 28 | | | | | | | exons 23x2, 32 | | | | | exon 20 | | exon 12 | | | |
| 9 | 9267 | GB | carcinoma | 3 | surgical specimen | F | | unknown | 12 | YES | | | | exon 7 | | | | | | | | | | | | exon 10 | | | | exon 16 | |
| 10 | 9268 | GB | adenocarcinoma | 2 | surgical specimen | F | 51 | NO | 12 | YES | | | exon 4 | exons 26, 27, 39 | | exons 10, 14 | exon 21 | exon 44 | exon 40 | | | | | exon 8 | exon 4 | | | | exon 5 | exon 16 | |
| 11 | 9270 | BILIARY | adenocarcinoma | 2 | surgical specimen | F | 54 | YES | 12 | NO | | | | | | | | ex 1 | ex 21 | | | ex 10 | | | | | ex 6, 14 | | | | |
| 12 | 9272 | GB | adenosquamous carcinoma | 3 | percutaneous biopsy | M | 72 | unknown | 2 | NO | | | | ex4 | | | | | | | ex4 | | | ex10 | | | | | | | |
| 13 | 9277 | GB | adenocarcinoma | 2 | surgical specimen | F | 78 | unknown | 37,66+ | YES | | | | | | | | ex16 | | | | | | | | | | | | | |
| 14 | 9278 | GB | adenosquamous carcinoma | 3 | surgical specimen | F | 75 | unknown | 7 | NO | | | | | | | ex11 | | | | | | | | | | | | | | |
| 15 | 9282 | GB | adenocarcinoma | 2 | surgical specimen | F | 75 | YES | 1 | NO | | | | | | | | | | | | | | | | | | | | | |
| 16 | 9279 | GB | adenocarcinoma | 3 | surgical specimen | F | 73 | unknown | 1 | NO | ex16 | | | ex13 | | | | ex13 | ex26 | | | ex19 | | | | | | | | | |
| 17 | 9280 | GB | adenocarcinoma | 2 | surgical specimen | F | 70 | YES | 13 | NO | | | | | | | | | | | | | | | | | | | | | |
| 18 | 9286 | GB | papillary carcinoma | | surgical specimen | F | 57 | unknown | 86+ | YES | | | | | | | | | ex34 | | | | | | | | | | | ex4x2 | |
| 19 | 9285 | GB | adenocarcinoma | 2 | surgical specimen | F | 59 | YES | 12 | YES | | | | | | | | | | | | | | | | | | | | ex4 | |
| 20 | | | | | | | | | | | | | | | | | | | | | | | | | | | | | | | |

| # | A | B | C | D | E | F | G | H | I | J | K | L | M | N | O | P | Q | R | S | T | U | V | W | X | Y | Z | AA | AB | AC | AD | AE |
|---|---|---|---|---|---|---|---|---|---|---|---|---|---|---|---|---|---|---|---|---|---|---|---|---|---|---|---|---|---|---|---|
| 1 | protocol number | diagnosis | histologic type | grade of differentiation | type of tissue | gender | age at diagn | lithiasis | OS | chemo | MSH2 | MSH3 | MSH6 | MTOR | MYC | NBN | NBPF1 | NCOR1 | NF1 | NF2 | NOTCH2 | NPEPPS | NRAS | NRG1 | NTRK1 | NTRK2 | NTRK3 | PALB2 | PARK2 | PBRM1 | PCNA |
| 2 | 9300 | GB | adenocarcinoma | 3 | surgical specimen | M | 61 | YES | 32+ | YES | | | | | | | | ex9 | ex55 | ex6 | ex27 | | ex4 | | | ex10 | ex5 | | | | |
| 3 | 9302 | GB | adenocarcinoma | 1 | surgical specimen | F | 61 | NO | 2+ | YES | | ex15 | ex4 | | | | | ex1,13,30 | | ex15 | ex31 | | | ex2 | | | | | | ex20,29 | ex6 |
| 4 | 9304 | GB | adenocarcinoma | 2 | surgical specimen | F | 62 | YES | 4+ | YES | | | | | | | | | | ex14 | ex25x2 | | | | | | | | | | |
| 5 | 9287 | GB | adenocarcinoma | 2 | surgical specimen | F | 63 | YES | 49.5 | YES | | | | | | | ex18 | | | | | | | | | | | | | | |
| 6 | 9297 | GB | adenocarcinoma | 2 | surgical specimen | F | 54 | YES | 8 | NO | | | | ex46 | ex2 | | | ex 25,39 | | ex3 | ex34 | in 17/18 | | | | | | | | | |
| 7 | 9295 | GB | adenocarcinoma | 2 | surgical specimen | M | 54 | YES | 9,5+ | YES | | ex2 | | | | | | ex39 | ex21 | | | | | | | ex7 | | | | | |
| 8 | 9296 | GB | adenocarcinoma | 2 | percutaneous biopsy | F | 37 | YES | 11 | NO | | | | ex54 | | | | | ex37 | | ex14 | | | | | | | | | ex29 | |
| 9 | 9299 | GB | adenocarcinoma | 2 | surgical specimen | F | 56 | YES | 19+ | YES | | | | ex28 | | | | | | | | | | | | | | | | | |
| 10 | 10090 | GB | adenocarcinoma | 2 | surgical specimen | F | 60 | YES | 19.5 | YES | | | | | | | | ex44 | | | ex31 | | | | | | | | | | |
| 11 | 10091 | GB | adenocarcinoma | 2 | surgical specimen | F | 62 | YES | 2.8 | YES | | | | | | | | | | | | | | | | | | | | | |
| 12 | 10092 | GB | adenocarcinoma | 2 | surgical specimen | F | 37 | YES | 21 | YES | | | | | | | | | | | | | | | | | | | | | |
| 13 | 10093 | GB | adenocarcinoma | 2 | surgical specimen | F | 46 | YES | 5 | YES | | | | | | ex7 | | | | | ex12 | | | | | | | | | ex16,20 | |
| 14 | | | | | | | | | | | | | | | | | | | | | | | | | | | | | | | |
| 15 | VARIANTS | | | | | | | | | | 1 | 3 | 2 | 7 | 1 | 2 | 3 | 9 | 8 | 4 | 9 | 4 | 1 | 4 | 1 | 4 | 2 | 2 | 1 | 7 | 1 |
| 16 | | | | | | | | | | | | | | | | | | | | | | | | | | | | | | | |
| 17 | GB=gallbladder cancer | | | | | | | | | | | | | | | | | | | | | | | | | | | | | | |
| 18 | M=male | | | | | | | | | | | | | | | | | | | | | | | | | | | | | | |
| 19 | F=female | | | | | | | | | | | | | | | | | | | | | | | | | | | | | | |
| 20 | NA=not available | | | | | | | | | | | | | | | | | | | | | | | | | | | | | | |

| | A | B | C | D | E | F | G | H | I | J | K | L | M | N | O | P | Q | R | S | T | U | V | W | X | Y | Z | AA | AB | AC | AD | AE |
|---|---|---|---|---|---|---|---|---|---|---|---|---|---|---|---|---|---|---|---|---|---|---|---|---|---|---|---|---|---|---|---|
| 1 | protocol number | diagnosis | histologic type | grade of differentiation | type of tissue | gender | age at diagn | lithiasis | OS | chemo | PDE4DIP | PDPR | PEG3 | PI3KCA | PIK3C2A | PIK3R1 | PIK3R5 | PIK3R6 | PLK1 | PMS2 | POLK | POU2F1 | PTCH1 | PTEN | PTPN11 | RADIL | RAD18 | RAD51 | RBBP8 | RB1 | RET |
| 2 | 9301 | GB | adenosquamous carcinoma | unknown | surgical specimen | M | 40 | NO | 3+ | NA | | | | | | | | | | | | | | | | | | | | | |
| 3 | 9046 | GB | adenocarcinoma | not reported | surgical specimen | F | 39 | YES | 15.33 | YES | | | exon 9 | | | exon 7 | | | | | | | | | | | | | | | |
| 4 | 9042 | GB | adenocarcinoma | 3 | surgical specimen | F | 66 | YES | 16 | YES | | | | | | | | | | | | | | | | | | | | | exon 8 |
| 5 | 9040 | GB | adenocarcinoma | 2 | surgical specimen | M | 58 | YES | 2.66 | YES | | | | | | | | | | | exon 7 | | | | | exon 3 | | | | | |
| 6 | 9259 | GB | adenocarcinoma | 2 | surgical specimen | F | 47 | NO | 4+ | YES | | | | | | | | | | | exon 3 | | | exon 5 | | | | | | exon 18 | |
| 7 | 9261 | GB | squamous carcinoma | 2 | surgical specimen | F | 73 | unknown | 30 | YES | | | | | | | | | | | | | | | | | | | | | |
| 8 | 9266 | GB | adenocarcinoma | 2 | surgical specimen | F | 57 | YES | 87 | YES | | | exon 9 | | | | | exon 4 | | | | exon 15 | | | exon 4 | exon 2 | | | | | |
| 9 | 9267 | GB | carcinoma | 3 | surgical specimen | F | | unknown | 12 | YES | exon 17 | | | in 18/19 | | | | | exon 6 | | | | | | | | | | | | exon 15 |
| 10 | 9268 | GB | adenocarcinoma | 2 | surgical specimen | F | 51 | NO | 12 | YES | exon 36 | exon 18 | | | exons 2, 16 | | exon 14 | | | | | | | | | | | | | | exon 7 |
| 11 | 9270 | BILIARY | adenocarcinoma | 2 | surgical specimen | F | 54 | YES | 12 | NO | | ex 19 | ex 9 | | | | | | | | | | | | | | | | | | |
| 12 | 9272 | GB | adenosquamous carcinoma | 3 | percutaneous biopsy | M | 72 | unknown | 2 | NO | | | | ex10; in15/16 | | | | | | | | | ex20 | | | | | | | | |
| 13 | 9277 | GB | adenocarcinoma | 2 | surgical specimen | F | 78 | unknown | 37,66+ | YES | | | | | | | | | | | | | ex18 | | | | | | | | |
| 14 | 9278 | GB | adenosquamous carcinoma | 3 | surgical specimen | F | 75 | unknown | 7 | NO | | | | | | | | | | | ex7 | | ex22 | | | | | | | | |
| 15 | 9282 | GB | adenocarcinoma | 2 | surgical specimen | F | 75 | YES | 1 | NO | | | ex9 | exon 2, ex7 | | | | | | | | | | | | | | | | | ex11 |
| 16 | 9279 | GB | adenocarcinoma | 3 | surgical specimen | F | 73 | unknown | 1 | NO | | | | | | | | | | | | | ex15 | | | | | | | | |
| 17 | 9280 | GB | adenocarcinoma | 2 | surgical specimen | F | 70 | YES | 13 | NO | | | | | | | | | | | | | | | | | | | | | |
| 18 | 9286 | GB | papillary carcinoma | | surgical specimen | F | 57 | unknown | 86+ | YES | | | | | | exon 8 | | | | | | | | | | | | | | | |
| 19 | 9285 | GB | adenocarcinoma | 2 | surgical specimen | F | 59 | YES | 12 | YES | | | | | | | | | ex10 | | | | ex22 | | | | | | | | |
| 20 | | | | | | | | | | | | | | | | | | | | | | | | | | | | | | | |

| | A | B | C | D | E | F | G | H | I | J | K | L | M | N | O | P | Q | R | S | T | U | V | W | X | Y | Z | AA | AB | AC | AD | AE |
|---|---|---|---|---|---|---|---|---|---|---|---|---|---|---|---|---|---|---|---|---|---|---|---|---|---|---|---|---|---|---|---|
| 1 | protocol number | diagnosis | histologic type | grade of differentiation | type of tissue | gender | age at diagn | lithiasis | OS | chemo | PDE4DIP | PDPR | PEG3 | PI3KCA | PIK3C2A | PIK3R1 | PIK3R5 | PIK3R6 | PLK1 | PMS2 | POLK | POU2F1 | PTCH1 | PTEN | PTPN11 | RADIL | RAD18 | RAD51 | RBBP8 | RB1 | RET |
| 2 | 9300 | GB | adenocarcinoma | 3 | surgical specimen | M | 61 | YES | 32+ | YES | ex13 | | | | | | ex17 | | ex5 | | | in 5/6, ex16 | ex3,8,10,17 | | | | | | | | |
| 3 | 9302 | GB | adenocarcinoma | 1 | surgical specimen | F | 61 | NO | 2+ | YES | ex23 | ex10 | ex9x2 | | ex18 | | | | | | | | | | | | | | ex17 | | |
| 4 | 9304 | GB | adenocarcinoma | 2 | surgical specimen | F | 62 | YES | 4+ | YES | | | | | | | | | ex5 | | | | | | | | | | | | |
| 5 | 9287 | GB | adenocarcinoma | 2 | surgical specimen | F | 63 | YES | 49.5 | YES | | | | | | | | | | | | | | exon 6 | | ex2 | | | | | |
| 6 | 9297 | GB | adenocarcinoma | 2 | surgical specimen | F | 54 | YES | 8 | NO | ex17 | | | | | | | | | | ex3 | in 15/16 | | | | | | | | | |
| 7 | 9295 | GB | adenocarcinoma | 2 | surgical specimen | M | 54 | YES | 9,5+ | YES | | | ex9x2 | | | | | | | | | | | | | | | | | | |
| 8 | 9296 | GB | adenocarcinoma | 2 | percutaneous biopsy | F | 37 | YES | 11 | NO | ex11 | | | | | | | | | | | | | | | | | | ex18 | | |
| 9 | 9299 | GB | adenocarcinoma | 2 | surgical specimen | F | 56 | YES | 19+ | YES | | | | | | | | | | | | | | | | | | | | | |
| 10 | 10090 | GB | adenocarcinoma | 2 | surgical specimen | F | 60 | YES | 19.5 | YES | | ex19 | | | | | | ex4 | | | | | | | | ex6 | | | | | |
| 11 | 10091 | GB | adenocarcinoma | 2 | surgical specimen | F | 62 | YES | 2.8 | YES | | | | | | | | | | | | | | | | | | | | | |
| 12 | 10092 | GB | adenocarcinoma | 2 | surgical specimen | F | 37 | YES | 21 | YES | | | | | | | | | | | | | | | | | | | | | |
| 13 | 10093 | GB | adenocarcinoma | 2 | surgical specimen | F | 46 | YES | 5 | YES | | | | | | | | | ex10 | | | | ex18 | | | | | | | | |
| 14 | | | | | | | | | | | | | | | | | | | | | | | | | | | | | | | |
| 15 | VARIANTS | | | | | | | | | | 6 | 4 | 6 | 3 | 2 | 2 | 2 | 2 | 5 | 0 | 4 | 3 | 7 | 2 | 1 | 4 | 0 | 0 | 2 | 1 | 4 |
| 16 | | | | | | | | | | | | | | | | | | | | | | | | | | | | | | | |
| 17 | GB=gallbladder cancer | | | | | | | | | | | | | | | | | | | | | | | | | | | | | | |
| 18 | M=male | | | | | | | | | | | | | | | | | | | | | | | | | | | | | | |
| 19 | F=female | | | | | | | | | | | | | | | | | | | | | | | | | | | | | | |
| 20 | NA=not available | | | | | | | | | | | | | | | | | | | | | | | | | | | | | | |

| | protocol number | diagnosis | histologic type | grade of differentiation | type of tissue | gender | age at diagn | lithiasis | OS | chemo | RFC1 | RNF43 | ROBO2 | ROS1 | RPA1 | RP11-145E5.5 | RPTOR | SBK1 | SETD1B | SETD2 | SFMBT1 | SF3B1 | SLX4 | SMAD 4 | SMARCA1 | SMARCAB1 | SMO | STK11 | TERT | TET1 | TET2 |
|---|---|---|---|---|---|---|---|---|---|---|---|---|---|---|---|---|---|---|---|---|---|---|---|---|---|---|---|---|---|---|---|
| 2 | 9301 | GB | adenosquamous carcinoma | unknown | surgical specimen | M | 40 | NO | 3+ | NA | | | | | | | | | | | | | | | | | | | | | |
| 3 | 9046 | GB | adenocarcinoma | not reported | surgical specimen | F | 39 | YES | 15.33 | YES | | | exon 15 | | | | | | | | | | | | | | exon 12 | | | | |
| 4 | 9042 | GB | adenocarcinoma | 3 | surgical specimen | F | 66 | YES | 16 | YES | | | | | | exon 1 | | | | | | | | | | | | | | | |
| 5 | 9040 | GB | adenocarcinoma | 2 | surgical specimen | M | 58 | YES | 2.66 | YES | | | | | | | | | | | | | | exon 5 | | | | | | exon 5 | |
| 6 | 9259 | GB | adenocarcinoma | 2 | surgical specimen | F | 47 | NO | 4+ | YES | | | | | | | | exon 4 | | exon 3 | | | | | | | | | | | |
| 7 | 9261 | GB | squamous carcinoma | 2 | surgical specimen | F | 73 | unknown | 30 | YES | | | | | | | | | exon 6 | | exon 4 | | | | | | | | | | |
| 8 | 9266 | GB | adenocarcinoma | 2 | surgical specimen | F | 57 | YES | 87 | YES | | | | exon 6 | exon 17 | | exon 20 | | exon 6 | exon 15 | | | | exon 4 | exon 18 | exon 5 | | exon 8 | | | |
| 9 | 9267 | GB | carcinoma | 3 | surgical specimen | F | | unknown | 12 | YES | | | | | | | | | in 8/9 | | | | | | | | exon 4 | | exon 2 | exon 11 | |
| 10 | 9268 | GB | adenocarcinoma | 2 | surgical specimen | F | 51 | NO | 12 | YES | | exon 7 | exons 16, 23 | | | | | | exon 2 | | | exon 24 | exons 2, 12 | | | | | exon 6 | | | |
| 11 | 9270 | BILIARY | adenocarcinoma | 2 | surgical specimen | F | 54 | YES | 12 | NO | | | | | | | | | | | | | | | | | | | | | |
| 12 | 9272 | GB | adenosquamous carcinoma | 3 | percutaneous biopsy | M | 72 | unknown | 2 | NO | | | | | | | | | | ex3 | | | | ex2 | | | | | | | |
| 13 | 9277 | GB | adenocarcinoma | 2 | surgical specimen | F | 78 | unknown | 37,66+ | YES | | | | | | | | | | | | | | | | | | exon 8 | | | |
| 14 | 9278 | GB | adenosquamous carcinoma | 3 | surgical specimen | F | 75 | unknown | 7 | NO | | | | | | | | | | | | | | ex4 | | | | | | | |
| 15 | 9282 | GB | adenocarcinoma | 2 | surgical specimen | F | 75 | YES | 1 | NO | | | | | | | | | | | | | ex2 | | | | | | | | |
| 16 | 9279 | GB | adenocarcinoma | 3 | surgical specimen | F | 73 | unknown | 1 | NO | | | | | | | | | ex13 | | | | | | | | | | | | ex 3, 11 |
| 17 | 9280 | GB | adenocarcinoma | 2 | surgical specimen | F | 70 | YES | 13 | NO | | | ex12 | | | | | | | | | | | | | | | | | | |
| 18 | 9286 | GB | papillary carcinoma | | surgical specimen | F | 57 | unknown | 86+ | YES | ex11 | | | | | | ex15 | | | | | | | | | | | | | | |
| 19 | 9285 | GB | adenocarcinoma | 2 | surgical specimen | F | 59 | YES | 12 | YES | | | | ex4 | | | | | | | | | | ex2 | | | | | | | |
| 20 | | | | | | | | | | | | | | | | | | | | | | | | | | | | | | | |

| | protocol number | diagnosis | histologic type | grade of differentiation | type of tissue | gender | age at diagn | lithiasis | OS | chemo | RFC1 | RNF43 | ROBO2 | ROS1 | RPA1 | RP11-145E5.5 | RPTOR | SBK1 | SETD1B | SETD2 | SFMBT1 | SF3B1 | SLX4 | SMAD 4 | SMARCA1 | SMARCAB1 | SMO | STK11 | TERT | TET1 | TET2 |
|---|---|---|---|---|---|---|---|---|---|---|---|---|---|---|---|---|---|---|---|---|---|---|---|---|---|---|---|---|---|---|---|
| 2 | 9300 | GB | adenocarcinoma | 3 | surgical specimen | M | 61 | YES | 32+ | YES | ex22 | | | | | | ex17 | | | exon 6, ex3 | | ex2,15 | | | ex22 | | | ex8 | | ex12 | |
| 3 | 9302 | GB | adenocarcinoma | 1 | surgical specimen | F | 61 | NO | 2+ | YES | ex17 | | | | ex14 | ex4 | | | ex12,15,16; in16/17 | ex7 | ex6 | | ex12,14 | | ex11 | | ex4 | | | | ex3 |
| 4 | 9304 | GB | adenocarcinoma | 2 | surgical specimen | F | 62 | YES | 4+ | YES | | | | | | | | | | | | | | | ex11 | | | | | | |
| 5 | 9287 | GB | adenocarcinoma | 2 | surgical specimen | F | 63 | YES | 49.5 | YES | | | | ex17 | | ex1 | | | | | | ex25 | | | | | | | | | |
| 6 | 9297 | GB | adenocarcinoma | 2 | surgical specimen | F | 54 | YES | 8 | NO | | ex43 | ex18 | | | | | | ex5,7 | | | | | | | | | | | | |
| 7 | 9295 | GB | adenocarcinoma | 2 | surgical specimen | M | 54 | YES | 9,5+ | YES | | | | | | | | | | | | | | | ex10 | | | | | | |
| 8 | 9296 | GB | adenocarcinoma | 2 | percutaneous biopsy | F | 37 | YES | 11 | NO | | | | | | | | | | | | | | | | | | | | | |
| 9 | 9299 | GB | adenocarcinoma | 2 | surgical specimen | F | 56 | YES | 19+ | YES | | | | | | | | | ex6 | | | ex8 | | | | | | | | ex11 | |
| 10 | 10090 | GB | adenocarcinoma | 2 | surgical specimen | F | 60 | YES | 19.5 | YES | | | ex15 | | | | | | | ex3 | | | ex10 | | | | | | | | |
| 11 | 10091 | GB | adenocarcinoma | 2 | surgical specimen | F | 62 | YES | 2.8 | YES | | | | | | | | | ex7 | | | | | | | | | | | | ex10 |
| 12 | 10092 | GB | adenocarcinoma | 2 | surgical specimen | F | 37 | YES | 21 | YES | ex13 | | | ex25 | | | | ex2 | | | | | | ex8 | | | | | | | |
| 13 | 10093 | GB | adenocarcinoma | 2 | surgical specimen | F | 46 | YES | 5 | YES | | | | | ex14 | | | | | | | | | | | | | | | | |
| 14 | | | | | | | | | | | | | | | | | | | | | | | | | | | | | | | |
| 15 | VARIANTS | | | | | | | | | | 4 | 2 | 5 | 4 | 3 | 3 | 3 | 2 | 8 | 6 | 2 | 4 | 4 | 6 | 5 | 1 | 3 | 4 | 1 | 4 | 3 |
| 16 | | | | | | | | | | | | | | | | | | | | | | | | | | | | | | | |
| 17 | GB=gallbladder cancer | | | | | | | | | | | | | | | | | | | | | | | | | | | | | | |
| 18 | M=male | | | | | | | | | | | | | | | | | | | | | | | | | | | | | | |
| 19 | F=female | | | | | | | | | | | | | | | | | | | | | | | | | | | | | | |
| 20 | NA=not available | | | | | | | | | | | | | | | | | | | | | | | | | | | | | | |

| protocol number | diagnosis | histologic type | grade of differentiation | type of tissue | gender | age at diagn | lithiasis | OS | chemo | TET3 | TGFBR1 | TGFBR2 | TPTE2 | TP53 | TSC1 | TSC2 | UIMC1 | UPF3A | VGLL4 | WEE1 | XIRP2 | YEATS4 | | variants per tumor |
|---|---|---|---|---|---|---|---|---|---|---|---|---|---|---|---|---|---|---|---|---|---|---|---|---|
| 9301 | GB | adenosquamous carcinoma | unknown | surgical specimen | M | 40 | NO | 3+ | NA | | | | | | | exon 13, 19 | | | | | | | | 4 |
| 9046 | GB | adenocarcinoma | not reported | surgical specimen | F | 39 | YES | 15.33 | YES | | | | | | exon 23 | | | | | | | | | 17 |
| 9042 | GB | adenocarcinoma | 3 | surgical specimen | F | 66 | YES | 16 | YES | | | | | | | | | | | | | | | 5 |
| 9040 | GB | adenocarcinoma | 2 | surgical specimen | M | 58 | YES | 2.66 | YES | | | | | | | | | | | | | | | 10 |
| 9259 | GB | adenocarcinoma | 2 | surgical specimen | F | 47 | NO | 4+ | YES | | | | | | | | | | | | | | | 10 |
| 9261 | GB | squamous carcinoma | 2 | surgical specimen | F | 73 | unknown | 30 | YES | | | | | | | | | | | | exon 4 | | | 14 |
| 9266 | GB | adenocarcinoma | 2 | surgical specimen | F | 57 | YES | 87 | YES | | | | | | exon 15 | exons 21, 35 | | | | | | | | 40 |
| 9267 | GB | carcinoma | 3 | surgical specimen | F | | unknown | 12 | YES | | | | | | exon 7 | | | | | | exon 7 | | | 29 |
| 9268 | GB | adenocarcinoma | 2 | surgical specimen | F | 51 | NO | 12 | YES | | exon 9 | | | | | | exon 5 | | ex2 | exon 11 | | | | 44 |
| 9270 | BILIARY | adenocarcinoma | 2 | surgical specimen | F | 54 | YES | 12 | NO | | | | | | | | | | | | | | | 13 |
| 9272 | GB | adenosquamous carcinoma | 3 | percutaneous biopsy | M | 72 | unknown | 2 | NO | | | | | exon 3,5 | | | | | | ex11 | | | | 16 |
| 9277 | GB | adenocarcinoma | 2 | surgical specimen | F | 78 | unknown | 37,66+ | YES | ex3 | | | | exon 2 | | | | | | | | ex3 | | 8 |
| 9278 | GB | adenosquamous carcinoma | 3 | surgical specimen | F | 75 | unknown | 7 | NO | | | | | exon 1 | ex7 | | | | | | | | | 11 |
| 9282 | GB | adenocarcinoma | 2 | surgical specimen | F | 75 | YES | 1 | NO | | | | | | | | | | | | | | | 9 |
| 9279 | GB | adenocarcinoma | 3 | surgical specimen | F | 73 | unknown | 1 | NO | ex7 | | | | ex1 | | | | | | | | | | 22 |
| 9280 | GB | adenocarcinoma | 2 | surgical specimen | F | 70 | YES | 13 | NO | | | | | exon 4 | | | | | | | | | | 5 |
| 9286 | GB | papillary carcinoma | | surgical specimen | F | 57 | unknown | 86+ | YES | | | | | | | | | | | | | | | 14 |
| 9285 | GB | adenocarcinoma | 2 | surgical specimen | F | 59 | YES | 12 | YES | | | exon 8 | | | | | | | | | | | | 11 |

| protocol number | diagnosis | histologic type | grade of differentiation | type of tissue | gender | age at diagn | lithiasis | OS | chemo | TET3 | TGFBR1 | TGFBR2 | TPTE2 | TP53 | TSC1 | TSC2 | UIMC1 | UPF3A | VGLL4 | WEE1 | XIRP2 | YEATS4 | | variants per tumor |
|---|---|---|---|---|---|---|---|---|---|---|---|---|---|---|---|---|---|---|---|---|---|---|---|---|
| 9300 | GB | adenocarcinoma | 3 | surgical specimen | M | 61 | YES | 32+ | YES | ex11 | | | | | ex10 | ex38 | | | | | ex7 | | | 36 |
| 9302 | GB | adenocarcinoma | 1 | surgical specimen | F | 61 | NO | 2+ | YES | ex3x2 | ex2x2,7 | | | exon 1 | | | | | | | ex3,7x2 | | | 51 |
| 9304 | GB | adenocarcinoma | 2 | surgical specimen | F | 62 | YES | 4+ | YES | | | | | | | ex35 | | | | | ex7 | | | 9 |
| 9287 | GB | adenocarcinoma | 2 | surgical specimen | F | 63 | YES | 49.5 | YES | | | | | ex2 | | exon 11, 14, 24 | | ex9 | | | ex7 | | | 22 |
| 9297 | GB | adenocarcinoma | 2 | surgical specimen | F | 54 | YES | 8 | NO | | | | | | | ex29 | | | | | | | | 20 |
| 9295 | GB | adenocarcinoma | 2 | surgical specimen | M | 54 | YES | 9,5+ | YES | | | ex6 | | exon 4 | | | | | | | | | | 16 |
| 9296 | GB | adenocarcinoma | 2 | percutaneous biopsy | F | 37 | YES | 11 | NO | ex9 | | | | | | | | | | | | | | 10 |
| 9299 | GB | adenocarcinoma | 2 | surgical specimen | F | 56 | YES | 19+ | YES | | ex4 | | | | | | | | | | | | | 11 |
| 10090 | GB | adenocarcinoma | 2 | surgical specimen | F | 60 | YES | 19.5 | YES | ex10 | | | | exon 1 | | | | | | | | | | 21 |
| 10091 | GB | adenocarcinoma | 2 | surgical specimen | F | 62 | YES | 2.8 | YES | | | | | | | | | | | | | | | 3 |
| 10092 | GB | adenocarcinoma | 2 | surgical specimen | F | 37 | YES | 21 | YES | | | | | | | ex33 | | | | | | | | 10 |
| 10093 | GB | adenocarcinoma | 2 | surgical specimen | F | 46 | YES | 5 | YES | | | | | | | | | | | | ex7 | | | 14 |
| VARIANTS | | | | | | | | | | 6 | 3 | 2 | 0 | 9 | 5 | 7 | 1 | 1 | 1 | 2 | 6 | 1 | | |

GB=gallbladder cancer
M=male
F=female
NA=not available
